# Modeling Virus-Induced Inflammation in Zebrafish: A Balance Between Infection Control and Excessive Inflammation

**DOI:** 10.3389/fimmu.2021.636623

**Published:** 2021-05-07

**Authors:** Con Sullivan, Brandy-Lee Soos, Paul J. Millard, Carol H. Kim, Benjamin L. King

**Affiliations:** ^1^ College of Arts and Sciences, University of Maine at Augusta, Bangor, ME, United States; ^2^ Department of Molecular and Biomedical Sciences, University of Maine, Orono, ME, United States; ^3^ Department of Environmental and Sustainable Engineering, University at Albany, Albany, NY, United States; ^4^ Department of Biomedical Sciences, University at Albany, Albany, NY, United States; ^5^ Department of Biological Sciences, University at Albany, Albany, NY, United States; ^6^ Graduate School of Biomedical Science and Engineering, University of Maine, Orono, ME, United States

**Keywords:** innate immunity, neutrophils, reactive oxidative species, inflammation, virus infection, zebrafish

## Abstract

The inflammatory response to viral infection in humans is a dynamic process with complex cell interactions that are governed by the immune system and influenced by both host and viral factors. Due to this complexity, the relative contributions of the virus and host factors are best studied *in vivo* using animal models. In this review, we describe how the zebrafish (*Danio rerio*) has been used as a powerful model to study host-virus interactions and inflammation by combining robust forward and reverse genetic tools with *in vivo* imaging of transparent embryos and larvae. The innate immune system has an essential role in the initial inflammatory response to viral infection. Focused studies of the innate immune response to viral infection are possible using the zebrafish model as there is a 4-6 week timeframe during development where they have a functional innate immune system dominated by neutrophils and macrophages. During this timeframe, zebrafish lack a functional adaptive immune system, so it is possible to study the innate immune response in isolation. Sequencing of the zebrafish genome has revealed significant genetic conservation with the human genome, and multiple studies have revealed both functional conservation of genes, including those critical to host cell infection and host cell inflammatory response. In addition to studying several fish viruses, zebrafish infection models have been developed for several human viruses, including influenza A, noroviruses, chikungunya, Zika, dengue, herpes simplex virus type 1, Sindbis, and hepatitis C virus. The development of these diverse viral infection models, coupled with the inherent strengths of the zebrafish model, particularly as it relates to our understanding of macrophage and neutrophil biology, offers opportunities for far more intensive studies aimed at understanding conserved host responses to viral infection. In this context, we review aspects relating to the evolution of innate immunity, including the evolution of viral pattern recognition receptors, interferons and interferon receptors, and non-coding RNAs.

## Introduction

Deadly hyperinflammatory responses to diseases like COVID-19 and influenza A result when the immune system overreacts (1–6). Cytokine storms induced by viral infections trigger this hyperinflammatory state, leading to serious consequences, including acute respiratory distress syndrome (ARDS), pulmonary edema, multiple organ failure, and death. The antiviral response encoded in vertebrate genomes incorporates an inflammatory rheostat (7) that is designed to ramp up or tamp down in response to infection. This response provides the host a measure of resilience and promotes its survivability. Under some circumstances, this inflammatory response to viral infection may become dysregulated, at which point an immunological tipping point is reached, leading to increased rates of mortality. This review describes progress in using the zebrafish (*Danio rerio*) as a powerful model system for the study of infection and inflammation, and it is increasingly being used to model human viral infections. Zebrafish possess several inherent characteristics that make them excellent biomedical and biological model systems, including optically clear embryos, high fecundity, a fully sequenced genome, amenability to multiple modes of injection and manipulation, and robust forward and reverse genetics tools. We review recent studies on viral recognition receptors in zebrafish that are homologous to those found on human cells. For example, we have shown that zebrafish possess α2,3- and α2-6-linked sialic acid receptors that are required for infection by certain influenza A virus (IAV) strains, including H1N1 (8). Because human viruses can infect zebrafish cells, it is possible to recapitulate aspects of the human viral disease in zebrafish, including the host inflammatory response. Many elements of the host immune response to human viral infection are retained in zebrafish, and this is owed to significant cellular and molecular conservation between zebrafish and humans. As neutrophils have critical roles in inflammation, we begin our review on neutrophils and their roles in antiviral response pathways that include toll-like receptors (TLRs), interferon (IFN) signaling, and the respiratory burst response. Next, we review zebrafish studies on fish and human viruses and include methodological details about these zebrafish models and functional assays. We also describe recent studies of non-coding RNAs that regulate neutrophil function. It is our view that the zebrafish offers tremendous promise as a model to understand how some of the mechanisms underlying a normal immune response to viral infection in humans become excessive, leading to increasing morbidities and mortalities.

## Immune Cell Conservation in Zebrafish

### Definitive Hematopoiesis

In zebrafish, definitive hematopoiesis begins as early as 26 hours post-fertilization (hpf) and gives rise to self-renewing hematopoietic stem cells (HSCs) that can differentiate into cells with myeloid, lymphoid, and erythroid lineages (9). The sites of definitive hematopoiesis differ between zebrafish and humans. For zebrafish, definitive hematopoiesis transitions from the ventral wall of the dorsal aorta (26 hpf) through the caudal hematopoietic tissue (CHT) (~2 days post-fertilization (dpf)) and eventually to the thymus (~3 dpf) or the pronephros/kidney (~4 dpf) (9–11). In mammals, definitive hematopoiesis is transitory as well, moving from the aorta-gonad-mesonephros region in the ventral wall of the dorsal aorta, to the mammalian fetal liver, and finally to the bone marrow (12). The earliest stage of definitive hematopoiesis in both zebrafish and mammals is restricted to analogous ventral dorsal aorta regions. From there, the anatomical sites of hematapoiesis differ (11). Nonetheless, the genetics and molecular signaling underlying definitive hematopoiesis in vertebrates are largely conserved across species. Importantly, the morphology and function of zebrafish neutrophils are conserved with mammalian neutrophils (13). As many studies of neutrophil function in zebrafish are done during embryonic and larval stages, it is worthwhile noting that neutrophils also arise from hematopoietic precursors in the yolk sac (14). As neutrophils are the first immune cells that migrate to the site of inflammation, our review will focus on these phagocytes.

### Neutrophils

The first immune cells that migrate to the site of inflammation are neutrophils (15). Zebrafish neutrophils, also known as heterophils, respond to infection and injury in a manner that is similar to human neutrophils. For example, zebrafish neutrophils have been shown to migrate to the sites of bacterial (16), fungal (17–23), and viral (8, 24, 25) infections. Additionally, wounding studies have demonstrated neutrophil migration to the site of injury in zebrafish (26). Like human neutrophils, the response of zebrafish neutrophils to pathogens include phagocytosis, degranulation, and formation of neutrophil extracellular traps (NETs). Central to the response of neutrophils is the release of reactive oxidative species (ROS), which is described in detail later in this review. Both azurophillic and non-azurophillic granules are found in zebrafish neutrophils, with azurophillic granules being more abundant (27, 28). Like primary azurophilic granules in mammalian neutrophils, zebrafish neutrophil granules contain the enzyme myeloperoxidase (Mpx) (27). During respiratory burst, Mpx catalyzes the conversion of H_2_O_2_ and Cl^-^ to produce cytoxic hypochlorous acid (HOCl) (29). Neutrophils also generate reactive nitrogen species (NO). NETs are released by neutrophils through a cell death process, named NETosis, to inactivate and destroy extracellular viral particles, bacteria, and fungi. In human neutrophils, NETs are composed of a scaffold of decondensed chromatin with at least 24 cytosolic and granule proteins, including myeloidperoxidase (MPO) and neutrophil elastase (ELANE) (30). NETs were observed to be generated by neutrophils found within whole zebrafish kidney tissue *ex vivo* following stimulation with calcium ionophore, phorbol myristate acetate (PMA), and β-glucan (31). Two features associated with NETs have been observed at the sites of localized hindbrain *Candida albicans* infection *in vivo*. First, increased levels of extracellular DNA were detected with neutrophil invasion following hindbrain *C. albicans* infection (32). Second, extrusion of a neutrophil-specific histone 2B-mCherry fusion protein was observed following neutrophil recruitment to *C. albicans* but not *C. auris* hindbrain infection (33). The activation and translocation of NETs is initiated by ROS that, in turn, stimulate MPO and ELANE expression in mammalian cells (34). Even though mammalian *ELANE* does not have an obvious homolog in zebrafish (35), elastase activity was associated with zebrafish NETs (31). Given the central role of ROS in the neutrophil response, a major focus in this review will be on ROS.

### Neutrophil and Macrophage Reporter Lines

Several zebrafish fluorescent reporter strains have been developed to visualize neutrophils and macrophages *in vivo*, and for fluorescently-activated cell sorting (FACS). Transgenic zebrafish neutrophil reporter lines have used *mpx* and lysozyme (*lyz*) promoters to drive the expression of fluorescent proteins. Frequently used neutrophil reporter lines include the GFP reporters, *Tg(mpx:GFP)^i114^* (36), *Tg(mpx:GFP)^uwm1^* (37) and *Tg(lyz:EGFP)^nz117^* (38), and the red fluorescent protein reporters, *Tg(mpx:mCherry)^uwm7^* (39) and *Tg(lyz:DsRED2)^nz50^* (38). Additional reporter lines using the photoconvertible fluorescent reporter, Dendra2 (40), have been developed to study migration of macrophages and neutrophils. Dendra2 protein photoswitches from green to red following exposure to visible blue or UV light. This photoconvertible reporter line enables tracking of neutrophil forward and reverse migration (41). Another photoconvertible protein, Kaede, has also been used to study neutrophil migration when expressed as part of a GAL4/UAS bipartite expression system, such as the *Tg(mpx:Gal4);Tg(UAS:Kaede)^i222^* line. As many zebrafish macrophage reporter lines have also been developed, it is possible to use double transgenic lines, such as *Tg(mpeg1:Gal4-VP16/UAS : Kaede/mpx:EGFP)*, to allow for *in vivo* imaging of neutrophils and macrophages simultaneously (42). These macrophage reporter lines use a promoter from the membrane attack complex/perforin-domain containing gene, macrophage expressed gene 1, tandem duplicate 1 (*mpeg1.1*) (43), to drive the expression of reporters, such as EGFP (*Tg(mpeg1:eGFP)^gl22^*) (42), mCherry (*Tg(mpeg1:mCherry)^gl23^*) (42), and YFP (*Tg(mpeg1:YFP)^w200^*) (44). Migration of macrophages can also be monitored using the Dendra2 reporter in the *Tg(mpeg1:Dendra2)^uwm12^* line (45). The promoter for microfibril associated protein 4, tandem duplicate 1 (*mfap4.1*) has also been used for macrophage reporter lines (46) as the expression of *mpeg1* was shown to be attenuated following infection of *Salmonella thyphimurium* and *Mycobacterium marinum* (47). Several of these neutrophil and macrophage reporter lines have been used for FACS for cell-specific functional analysis (38, 48, 49).

## Overview of Antiviral Response

**Figure 1:**
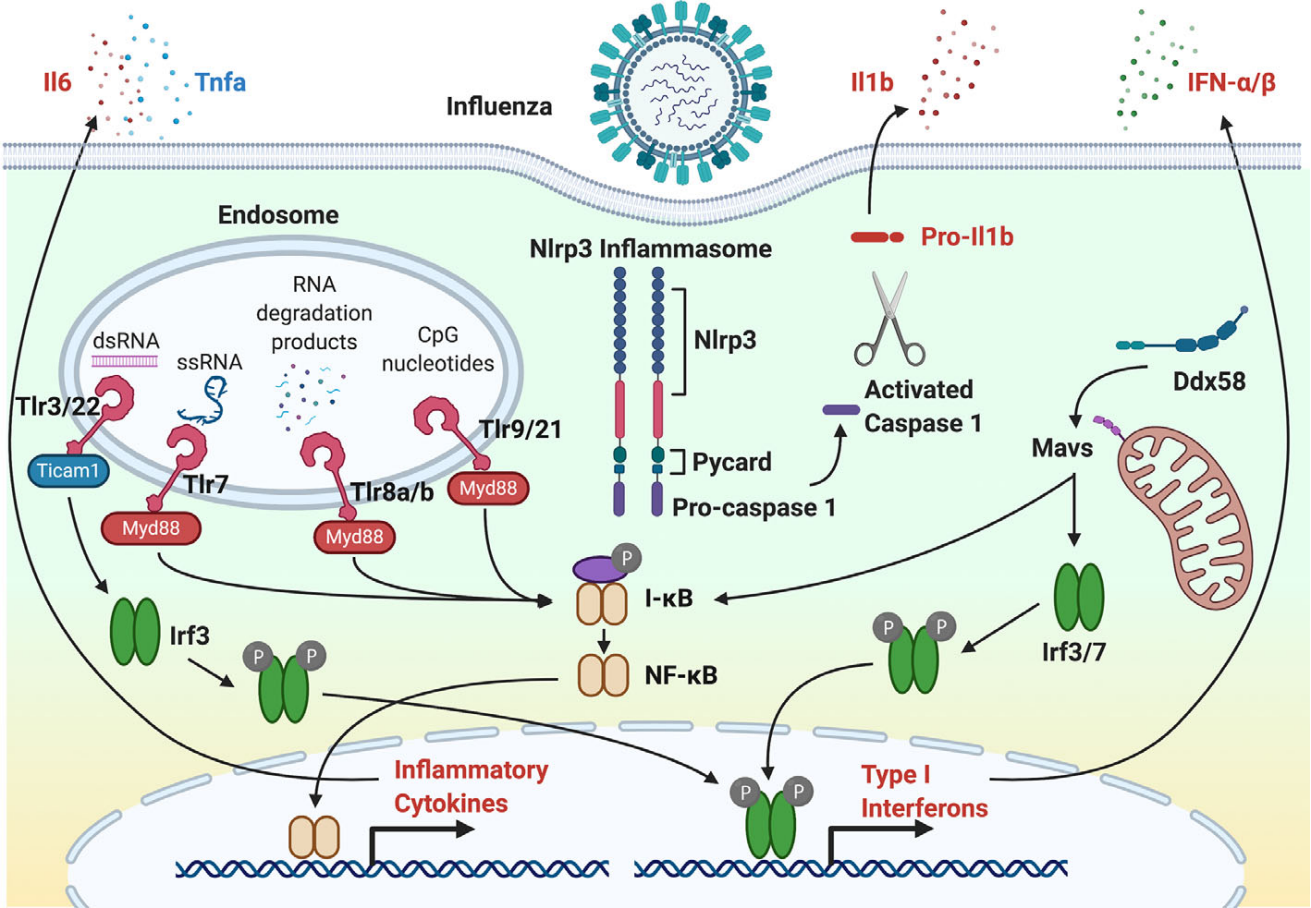
The antiviral response to Influenza A Virus infection. Following IAV entry and infection, single-stranded RNA (ssRNA) and RNA degradation products incorporated into endosomes are recognized by Tlr7 and Tlr8a/b, respectively. In other virus infections, double-stranded RNA by Tlr3 and Tlr22. CpG motifs are recognized and Tlr9 and Tlr21. For Tlr7, Tlr8a/b and Tlr9, the TLR-adaptor, Myd88, activates the NF-κB transcription factor through IkB. NF-κB initiates transcription of inflammatory cytokines, such as Il6, Il1b, and Tnfa. For Tlr3, the TLR-adapter, Ticam1, activates Irf3 that initiates transcription of type I interferons. DAMPs and PAMPs can activate the Nlrp3 inflammasome through activated caspase 1. Activation of RIG-I (Ddx58) by cytosolic viral RNA activates Irf3 and Irf7 transcription factors through Mavs. Irf3 and Irf7 initiate the expression of type 1 interferons that further exacerbates the antiviral innate immune response to infection.

Defense against viral infection is governed by both the innate and adaptive immune systems. Even though the adaptive immune system can provide protection from viral infection through B and T lymphocytes, the innate immune system provides an initial response to viral infection and is the focus of this review. The innate immune system includes physical barriers, phagocytic cells, pattern recognition receptors (PRRs), interferons and interferon-stimulated genes (ISGs), cytokines and chemokines, and the complement system. Physical barriers include the mucus barrier that is composed of polymeric secreted mucins. Phagocytes include neutrophils and macrophages that can kill virus particles and recruit additional phagocytes to sites of infection. An important response of phagocytes is a respiratory burst response that releases ROS to kill virus particles and recruit additional phagocytes. Critical to the activation of immune response are PRRs that bind pathogen-associated molecular patterns (PAMPs) and damage-associated molecular patterns (DAMPs) and trigger the expression of interferon and cytokines through NF-κB and interferon response factor (IRF) transcription factors. Interferon elicits a potent response to viral infection that includes the activation of a battery of ISGs. Inflammatory cytokines and chemokines recruit phagocytes at the site of infection. The complement system functions to respond to microbial pathogens by recognizing motifs through three convergent activation pathways that lead to complement-mediated lysis (50). **Figure 1** illustrates components of response to viral infection using IAV as an example. Genes that have shown to respond to the inflammatory and antiviral response using zebrafish models of viral infection are shown in **Tables 1** and **2**, respectively.

**Table 1:** Table of proinflammatory genes studied in zebrafish models of viral infection.

Gene Symbol	Example Viruses				
	Group V				
	Spring Viremia of Carp Virus (SVCV)	Tilapia Lake Virus (TLV)	Snakehead Rhabdovirus (SHRV)	Infectious Hematopoietic Necrosis Virus (IHNV)	Influenza A Virus (IAV)
*caspa*	(51)				
*cxcl8a*	(52)	(53)			(25)
*ifng1*	(54)	(53)			
*ifnphi1*	(51, 55, 56) # (57)	(53)	(58–60)	(61)	(8)
*ifnphi2*	(51)				
*il1b*	(51, 52, 54)	(53)			(25)
*irf3*	(52, 56, 62)	(53)			
*irf7*	(52, 62)	(53)			
*lta*	(52, 56)				
*sting1*	(62)				
*pycard*	(51)				
*rarres3*	(51)				
*tnfa*	(51, 54, 55)	(53)			(25)
*tnfb*	(55)				

^#^Functional study.

**Table 2:** Table of antiviral genes studied in zebrafish models of viral infection.

Gene Symbol	Example Viruses					
	Group III	Group V				
	Infectious Pancreatic Necrosis Virus	Spring Viremia of Carp Virus	Tilapia Lake Virus	Snakehead Rhabdovirus	Infectious Hematopoietic Necrosis Virus	Influenza A Virus
*defb2*		(54)				
*foxo3b*		(52)				
*ifit8*		(51)				
*ifit14*		(51)				
*isg15*	(61)	(61)			(61)	
*mavs*		(62, 63)				
*ifih1*		(63)		(60)		
*mxa*		(51, 54, 63)	(53)	(59)		(8)
*mxb*		(51, 54, 55)				
*mxc*		(52, 54, 56)				
*nod2*		(63)				
*pkz*		(56)				
*prmt3*		(56)				
*rela*		(64)				
*ddx58*		(51, 52, 62, 63)	(53)			
*ripk2*		(63)				
*tbk1*		(62)				
*tlr3*		(51)	(53)	(65)		
*tlr7*		(51)				
*tlr8a*		(51)				
*tlr22*		(51)	(53)			
*rsad2*		(55)				

The zebrafish model system holds particular promise for understanding the innate immune response to viral infection. Zebrafish lack a fully functional adaptive immune response for the first 4-6 weeks of development (66) and rely upon their innate immune response for defense against all forms of infection. Many aspects of the innate immune system, including those listed below, are functionally conserved in zebrafish, and thus the zebrafish can effectively model how normal inflammatory responses to viral infections can lead to extensive tissue damage and mortality.

### Pattern Recognition Receptors (PRRs)

PRRs bind PAMPs and DAMPs, triggering a signal transduction cascade that activates several transcription factors critical to the antiviral and pro-inflammatory immune response. Viral PAMPs include surface glycoproteins, single-stranded RNA, double-stranded RNA, and other RNA and DNA species. DAMPs produced by damaged cells can also activate the immune response. DAMPs include denatured intracellular proteins, such as high-mobility group box protein 1 (HMGB1) (67). PRRs include TLRs, nucleotide-binding oligomerization domain (NOD)-like receptors (NLRs), retinoic acid-inducible gene-I-like (RIG-I)-like receptors (RLRs), scavenger receptors, and C-type lectin receptors (CLRs).

PAMPs from viral particles that have entered the phagolysosomal degradation pathway are recognized by mammalian endosomal TLRs: TLR3, TLR7, TLR8 and TLR9. These TLRs traffic from the endoplasmic reticulum (ER) to endosomes with the chaperone, UNC93B1 (68). Double-stranded RNA, single-stranded RNA, RNA degradation products, and CpG-deoxynucleotides (CpG-DNA) are recognized by TLR3, TLR7, TLR8 (69) and TLR9, respectively. TLR3, TLR7, TLR8, and TLR9 are conserved in zebrafish as the homologs *tlr3* (65), *tlr7* (70), *tlr8a* (70), *tlr8b* (70), and *tlr9* (70, 71) (**Table 3**). In zebrafish, two additional antiviral TLRs, *tlr21* and *tlr22*, have been described that recognize CpG-DNA (71) and double-stranded RNA (73, 79), respectively. Homologs of *tlr21* and *tlr22* have not been observed in mammalian genomes, but *tlr21* is conserved in avian species.

**Table 3:** TLR genes in zebrafish.

Zebrafish Gene Symbol	Ensembl Zebrafish Gene ID	Zebrafish Chr.	Zebrafish Refs.	Predicted Human Ortholog	Ensembl Human Ortholog Gene ID	Human Chr.	Orthology Resource(s)
*tlr1*	ENSDARG00000100649	14	(70)	TLR1*	ENSG00000174125	4	Synteny DB, ZFIN
				TLR6*	ENSG00000174130	4	Ensembl
*tlr2*	ENSDARG00000037758	1	(70, 72)	*TLR2*	ENSG00000137462	4	Synteny DB, ZFIN, Ensembl
*tlr3*	ENSDARG00000016065	1	(65, 73, 74)	*TLR3*	ENSG00000164342	4	Synteny DB, ZFIN
*tlr4al*	ENSDARG00000075671	13		**			
*tlr4ba*	ENSDARG00000019742	13	(75)	**			
*tlr4bb*	ENSDARG00000022048	13	(75)	**			
*tlr5a*	ENSDARG00000044415	20	(72, 76)	*TLR5*	ENSG00000187554	1	Synteny DB, ZFIN
*tlr5b*	ENSDARG00000052322	20	(72, 76, 77)	*TLR5*	ENSG00000187554	1	Synteny DB, ZFIN
*tlr7*	ENSDARG00000068812	9	(70, 78)	*TLR7*	ENSG00000196664	X	Synteny DB, ZFIN
*tlr8a*	ENSDARG00000090119	KN150362.1	(70)	*TLR7*	ENSG00000196664	X	Synteny DB
*CU914164.1* (*tlr8*)	ENSDARG00000104832	9		*TLR8*	ENSG00000101916	X	ZFIN
*tlr8b*	ENSDARG00000073675	10	(70)	*TLR7*	ENSG00000196664	X	Synteny DB
				*TLR8*	ENSG00000101916	X	ZFIN
*tlr9*	ENSDARG00000044490	8	(71)	*TLR9*	ENSG00000239732	3	Synteny DB, ZFIN
*tlr18*	ENSDARG00000040249	16		–		– –	
*tlr19*	ENSDARG00000026663	16		–		–	
*tlr20.1*	ENSDARG00000115923	9		–		–	
*tlr20.2*	ENSDARG00000088701	9		–		–	
*tlr20.3*	ENSDARG00000114057	9		–		–	
*tlr21*	ENSDARG00000058045	16	(71)	–		–	
*tlr22*	ENSDARG00000104045	21	(73, 79)	–		–	

Predictions were made using information from Ensembl, ZFIN and SyntenyDB. Ensembl gene IDs for each gene are listed, along with their respective chromosome locations. *Synteny Database and ZFIN predict TLR1 as the human ortholog, while Ensembl predicts TLR6. In the human genome assembly, TLR1, TLR6, and TLR10 are directly adjacent to one another. **Human TLR4 is not orthologous to tlr4ba, tlr4bb, or tlr4al (75). Known zebrafish toll-like receptors with predicted human orthologs.

The TLR signaling pathway in zebrafish includes the adaptor proteins Myd88, Tirap, Ticam1, and Sarm1 for downstream signaling. The gene encoding the Ticam2 adaptor protein found in mammals is absent in zebrafish (74). In mammals, Myd88 is required for all TLRs except for TLR3 and TLR4 (80). TLR signaling is mediated by tumor necrosis factor receptor associated factor 6 (TRAF6) and interleukin-1 receptor-associated kinase 4 (IRAK4) that activate the NFκB, IRF, STAT, ATF, and AP-1 families of transcription factors. The expression of *tlr3*, *traf6* and *irak4* was upregulated in embryonic and adult zebrafish following snakehead rhabdovirus (SHRV) infection (65). Beyond these four TLRs, knockdown of two adaptors for TLR signaling, Ly86 and Cd180, found increased susceptibility to spring viremia carp virus (SVCV) in zebrafish larvae (81). In mammals, LY86 and CD180 are adaptors for TLR4, a TLR that responds to lipopolysaccharide (LPS).

We previously described a model for the history of *TLR4* genes in humans and zebrafish that we believe accounts for the functional divergence that has been observed, specifically in regards to the reduced LPS sensitivity seen in fishes (75). We hypothesize that *TLR4* was duplicated in an ancestral genome with the second whole genome duplication event, yielding the *TLR4A* and *TLR4B* genes (75). Our model projects that there was lineage divergence and a reciprocal loss of *TLR4* ohnologs. The ancestral *TLR4A* was retained in the lineage that gave rise to mammals, including humans, and *TLR4B* was lost. The *TLR4A* gene, by convention, is referred to as *TLR4*. In the lineage that gave rise to zebrafish, the ancestral *TLR4B* gene was retained, and the ancestral *TLR4A* gene was lost. The ancestral *TLR4B* gene was subsequently duplicated, giving rise to the *tlr4ba* and *tlr4bb* paralogs observed in the current zebrafish genome.

There are data that indicate that *TLR3*, *TLR7*, *TLR8*, and *TLR9* are, at least to some extent, functionally conserved in zebrafish as the homologs *tlr3, tlr7, tlr8a, tlr8b*, and *tlr9*. To fully exploit the zebrafish model as a means to understand antiviral responses, it is necessary to undertake meticulous gene history studies to support orthology. Indeed, based on data available through Ensembl (82), ZFIN (83), and the Synteny Database (84), there appear in certain instances to be discrepancies in the identification and/or naming of zebrafish *TLR* genes that consequently imply a gene orthology (or lack of orthology) and functional conservation with human *TLR* genes despite sufficient evidence. For example, ZFIN predicts that zebrafish *tlr8a* and *tlr8b* are co-orthologous to human *TLR8*; however, this prediction is not supported by Ensembl or the Synteny Database where they do not list any orthologs for human *TLR8*. According to Ensembl, zebrafish *tlr8b* has a one-to-many orthologous relationship to the spotted gar gene *ENSLOCG00000013826*, which has been annotated as *tlr3*. Due to its evolutionary position as a non-teleost and non-tetrapod, jawed vertebrate model organism, the spotted gar genome serves as an “orthology bridge” to link the gene histories of the zebrafish (and other teleosts) and human genomes (85). The *ENSLOCG00000013826* gene has no human ortholog but does have a one-to-many orthologous relationship to a zebrafish gene annotated as *tlr3*. According to Ensembl and the Synteny Database, this zebrafish *tlr3* gene is an ortholog to human *TLR3*. This brief example demonstrates the inconsistencies present in current zebrafish databases and lends credence to the idea that the *tlr8* paralogs found in zebrafish (and other fishes) have no ortholog in the human genome, and thus are likely misnamed. In addition to these issues related to the evolutionary history of zebrafish *tlr* genes, there are also important concerns about the mechanisms by which the proteins encoded by these genes are engaged. Specifically, there is evidence that zebrafish TLR proteins do not bind PAMPs and other ligands in the same manner as human TLR proteins (69). There is also evidence indicating that the mechanisms by which zebrafish TLR proteins engage TIR domain containing adaptor proteins may sometimes differ (74). There are also many questions related to where within or on a cell a zebrafish TLR protein is expressed. Taken together, it is clear that assumptions about zebrafish TLR protein function based upon protein similarity and even phylogenetic analyses need further verification through comprehensive gene history analysis and thorough validation through functional assays.

Cytosolic PAMPs and DAMPs are recognized by NLRs and RLRs. After ligand binding, two NLRs, NOD1 and NOD2, can activate NFκB after recruiting the serine/threonine kinase RIPK2 through MAP kinase signaling. Several NLRs, including NLRC4, NARP1 and NARP3, function as PAMP and DAMP receptors for inflammasomes. Inflammasomes are multiprotein complexes that activate inflammatory caspases and pro-inflammatory cytokines through canonical signaling and non-canonical pathways to induce pyroptosis (86). In the canonical NLRP3 inflammasome signaling pathway, ligand binding to NRLs activate caspase 1 (CASP1) that then then activates the pro-inflammatory cytokines, interleukin 1β (IL1B) and interleukin 18 (IL18). Activation of CASP1 is dependent on the adaptor protein, apoptosis-associated speck-like protein containing a caspase-recruitment domain (PYCARD), which is also part of the inflammasome complex. In the non-canonical NLRP3 inflammasome signaling pathway, activated inflammasomes hydrolyze gasdermin D (GSDMD) leading to a N-terminal fragment that perforates the cell membrane to enable the release cytokines and subsequent cell death through pyroptosis. Inflammasome NLRs recognize ligands from both infection and sterile stressors. NLRP3 recognizes double-stranded RNA and activates CASP1 after binding the adaptor protein, apoptosis-associated speck-like protein containing a caspase-recruitment domain (PYCARD). Pycard-dependent activation of Il1b by Nlrp3 inflammasomes through caspase 1 (*caspa*) was found to be conserved in zebrafish larvae using morpholino knockdown of Nlrp3 and a *nlrp3* mutant challenged with *Edwardsiella tarda* (87). Li et al. also showed Nlrp3 initiated cell pyroptosis through Caspb activation in a gasdermin E (Gsdmeb/Gsdmea)-dependent, but independent of Pycard-activation (87). While several aspects of inflammasome signaling are conserved in zebrafish, differences do exist. Zebrafish have over 400 NLR genes (88), but only two have been associated with inflammasome function, *nlrp1* (89), and *nlrp3* (87, 90), that were shown to function similar to NLRP1. An additional inflammasome adaptor, *caiap*, was found to regulate inflammasome activation in zebrafish in response to *Salmonella typhimurium* infection (91). While the pro-inflammatory cytokine, *il1b* is conserved with zebrafish, an ortholog to IL18 has not been identified in zebrafish. Homologs to IL18 have been identified in other ray-finned fishes, including the pufferfish (*Takifugu rubripes*) (92) and rainbow trout (*Oncorhynchus mykiss*) (93).

Cytosolic viral RNA can also be detected by RLRs that are a family of DExD/H box RNA helicases consisting of RIG-I (encoded by the gene *DDX58*), melanoma differentiation-associated factor 5 (MDA5; encoded by the gene *IFIH1*), and laboratory of genetics and physiology 2 (LGP2; encoded by the gene *DHX58*). Activation of RLRs by binding viral RNA leads to activation of the antiviral response and type 1 interferon (IFN) expression through interferon regulatory factor 3 (IRF3), IRF7, and NF-κB transcription factors. Upon binding viral RNA, the CARD domains of RIG-1 and MDA5 interact with the adaptor protein, mitochondrial antiviral signaling (MAVS). The conserved role of Mavs in regulating the IFN antiviral response in zebrafish larvae has been demonstrated through studies of chikungunya virus (CHIKV) infection (94). The IFN response and survival was significantly reduced in Mavs morphants infected with CHIKV. Zebrafish homologs of *DDX58*, *IFIH1* and *DHX58* have been identified as *ddx58*, *ifih1*, and *dhx58*.

Additional PAMP receptors include scavenger receptors and CLRs. In mammalian models, the scavenger receptor, macrophage receptor with collagenous structure (MARCO), has been shown to recognize several viruses, including respiratory syncytial virus and vaccinia virus. In zebrafish, *marco* has been used as a marker of macrophages and dendritic cells in adults. Marco was demonstrated to be required for phagocytosis and the proinflammatory response to *Mycobacterium marinum* and *Salmonella typhimurium* in larvae (95). Increased bacterial burden and decreased proinflammatory signaling was observed in infected Marco morphants. Another scavenger receptor, the expression of cluster differentiation antigen 36 (*cd36*) was upregulated in zebrafish following infection by viral hemorrhagic septicemia virus (VHSV) (96). Knockdown of Cd36 in zebrafish embryos resulted in higher bacterial burden following infection by *Mycobacterium marinum* (97). Several transmembrane CLR proteins function as PRRs on myeloid cells. Two CLRs include mannose-binding lectin 2 (MBL2) and CD209. MBL2 can activate the lectin complement pathway (98) after binding to mannose, fucose and N-acetylglucosamine on microbial pathogens, including viruses. MBL2 was shown to bind to influenza A virus (IAV) and inhibit the hemagglutinating activity of IAV (99). CD209 can also recognize microbial pathogens, including viruses that express mannose-rich oligosaccharides. CD209 was shown to function as an attachment receptor for influenza A virus on mammalian cells and mediate sialic-acid independent attachment and infection (100). While the functions of these specific CLRs have not yet been investigated in the context of viral infection in zebrafish, both *mbl2* and *cd209* are present in the zebrafish genome.

The complement system has important roles in innate immunity and neutralization of viruses. Mechanisms for complement activation include C-reactive protein (CRP), and recognition of PAMPs and DAMPs. The classical, lectin and alternative complement pathways activate C3 convertase that cleaves complement component C3 to produce the C3a and C3b peptides. In the alternative pathway, C5 convertase cleaves C5 to produce C5a and C5b. Both anaphylatoxin, C3a, and C5a have important roles in regulating inflammation (101). C3a inhibits the migration of neutrophils to sites of acute inflammation (102) whereas C5a has the opposite function (103). The complement system is largely conserved in zebrafish, but there are differences (50). For example, there are two groups of paralogs for C3, *c3a* with six paralogs (*c3a.1, c3a.2, c3a.3, c3a.4, c3a.5*, and *c3a.6*), and *c3b* with two paralogs (*c3b.1* and *c3b.2*), however there is only one C5 homolog, *c5*. A zebrafish study of CRP genes and proteins in the response to SVCV and VHSV infection showed that *crp2*/Crp2 and *crp5*/Crp5 had the largest increases in expression (104).

### Interferons and Interferon-Responsive Genes

The innate immune response to viral infection is governed by interferon (IFN) and genes induced by interferon. In mammals, there are three classes of interferon genes (IFNs): type I (α, β, ω, ε, and κ), type II (γ) and type III (λ). Both type I and type III IFNs have well established antiviral activities in mammals, whereas the function of type II IFNs is associated with the response to bacterial infection. Type II IFNs do not exclusively respond to bacterial infection, as they have been associated with the response to vesicular stomatitis virus infection in mice (105). Beyond the type I IFN genes discussed in detail below, zebrafish have two paralogs of the type II IFN, *IFNG*, named *ifng1* (interferon gamma 1) and *ifng1r* (interferon gamma 1 related) (106).

Activation of IFN is a conserved response to viral infection across vertebrates, including zebrafish. One of the first studies in zebrafish showed that IFN expression was induced in zebrafish liver cells when infected by SHRV (58). In addition to the IFN gene first characterized in that study (now named *ifnph1*), zebrafish have three additional IFN genes (*ifnphi2*, *ifnphi3*, *ifnphi4*) that are activated in response to viral infection (**Table 4**) (107, 108). Considerable efforts to identify and characterize IFN genes in fishes have been undertaken, and several excellent reviews describing the complexity of IFN signaling in fishes, including zebrafish, have recently been published (110–112). Type I IFN signaling mediated by zebrafish bears many similarities but also significantly differs from that observed in humans. For example, at the gene level, fish type I IFN (including zebrafish) have retained introns, while mammalian type I IFNs do not. It is thought that the absence of mammalian type I IFNs was a result of a retrotransposition event in amniotes (111). In addition, unlike mammalian type I IFNs, which are typically secreted upon viral induction, fish type I IFNs can be alternatively transcribed with or without signal peptides for extracellular expression (57). Zebrafish type I IFNs can be separated into two groups: Group I and Group II (111). Group I IFNs include Ifnphi1 and Ifnphi4, while group II IFNs include Ifnphi2 and Ifnphi3. Group I IFNs are characterized by a pair of conserved cysteine residues that form a disulfide bridge. Group II IFNs are characterized by two pairs of conserved cysteine residues that form two disulfide bridges (113). Group I and group II IFNs engage different receptor complexes, but each receptor complex is thought to include cytokine receptor family member b 5 (Crfb5) (108). Group I IFNs are thought to interact with Crfb1/Crfb5 complexes, and group II IFNs are thought to interact with Crfb2/Crfb5 complexes. Interestingly, knockdown of caveolin 1 (Cav1) in zebrafish disrupted Crfb1 IFN receptor clusters, thereby decreasing antiviral immune responses (114). Activation of the IFN receptor clusters signal through the Jak/STAT pathway to activate IFN-stimulated genes (ISGs) that share a IFN-stimulated response element (ISRE) (115). Multiple studies have shown a large set of ISGs in response to viral infection in zebrafish, many of which have mammalian orthologs that are ISGs in mammalian models. Among some of these conserved ISGs are *mxa* (116), *rsad2* (57), and *isg15* (61). One study compared ISGs that responded to a poor IFN inducer, infectious hematopoietic necrosis virus (IHNV), to a strong IFN inducer, CHIKV, with and without knockdown of the IFN receptors, Crfb1 and Crfb2 (117). A study of zebrafish infected with SVCV found that 382 and 926 genes were differentially expressed in brain and spleen, respectively (118). Given that ISGs have antiviral effects and, in some cases, also enhance the replication of viruses (115), more studies are needed to understand the complexity of IFN signaling.

**Table 4:** IFN genes in zebrafish.

Zebrafish Gene						Predicted Human Ortholog	Ensembl Human Gene ID	Orthology Resource(s)
Gene Symbol	Ensembl Gene ID	Chr.	IFN Type	Refs.	Role in Virus Infection			
*ifnphi1*	ENSDARG00000025607	3	Type I(Group 1)	(58)	SHRV (58–60)SVCV (51, 55, 56, 61)# (57)INHV (61)IAV (8)TLV (53)CHIKV# (94)			–
*ifnphi2*	ENSDARG00000069012	3	Type I (Group 2)	(107, 108)	SVCV (51)			–
*ifnphi3*	ENSDARG00000070676	3	Type I (Group 2)	(107, 108)				–
*ifnphi4*	ENSDARG00000100678	12	Type I (Group 1)	(107, 108)	SVCV (108) INHV (108)			–
*ifng1*	ENSDARG00000024211	4	Type II	(106)	SVCV (54, 109) TLV (53)	*IFNG*	ENSG00000111537	ZFIN, Ensembl
*ifng1r*	ENSDARG00000045671	4	Type II	(106)		*IFNG*	ENSG00000111537	ZFIN, Ensembl

^#^Functional study.

In zebrafish, the *ifnphi1* gene can express two transcript isoforms: a longer, constitutively-expressed transcript, which lacks sequence encoding a secretion signal peptide, and thus is likely retained within the cells, and a shorter, virally-induced transcript, which contains a signal peptide that causes the protein to be secreted (57). Transcripts encoded by the *ifnphi1* gene also exhibit discrete spatiotemporal patterns (108). Basal levels of *ifnphi1* are elevated in adult spleens relative to whole larvae. In both adult and larval fish, viral infection could induce increased expression levels. Using the transgenic zebrafish line *Tg(ifnphi1:mCherry)*, Palha et al. (94) showed expression of mCherry fluorescent protein driven by the *ifnphi1* promoter in hepatocytes and neutrophils following infection with CHIKV. Transcripts encoded by the *ifnphi2* gene were below the level of detection in larval zebrafish and were expressed levels comparable to *ifnphi1* in adult spleens (108). In adult fish, splenic expression of *ifnphi2* transcripts could be induced by SVCV infection. Transcripts encoded by the *ifnphi3* gene are expressed at elevated basal levels in both adult spleens and whole larvae and were not induced by SVCV or IHNV infection (108). Interestingly, expression of *ifnphi3* transcripts were not observed in the same cells in a *ifnphi3* promoter reporter transgenic fish, although these data were shared as part of a personal communication and were not yet published (111). Transcripts encoded by the *ifnphi4* gene are expressed at modest basal levels and are mildly induced by SVCV in larvae (108).

## Respiratory Burst Response

**Figure 2:**
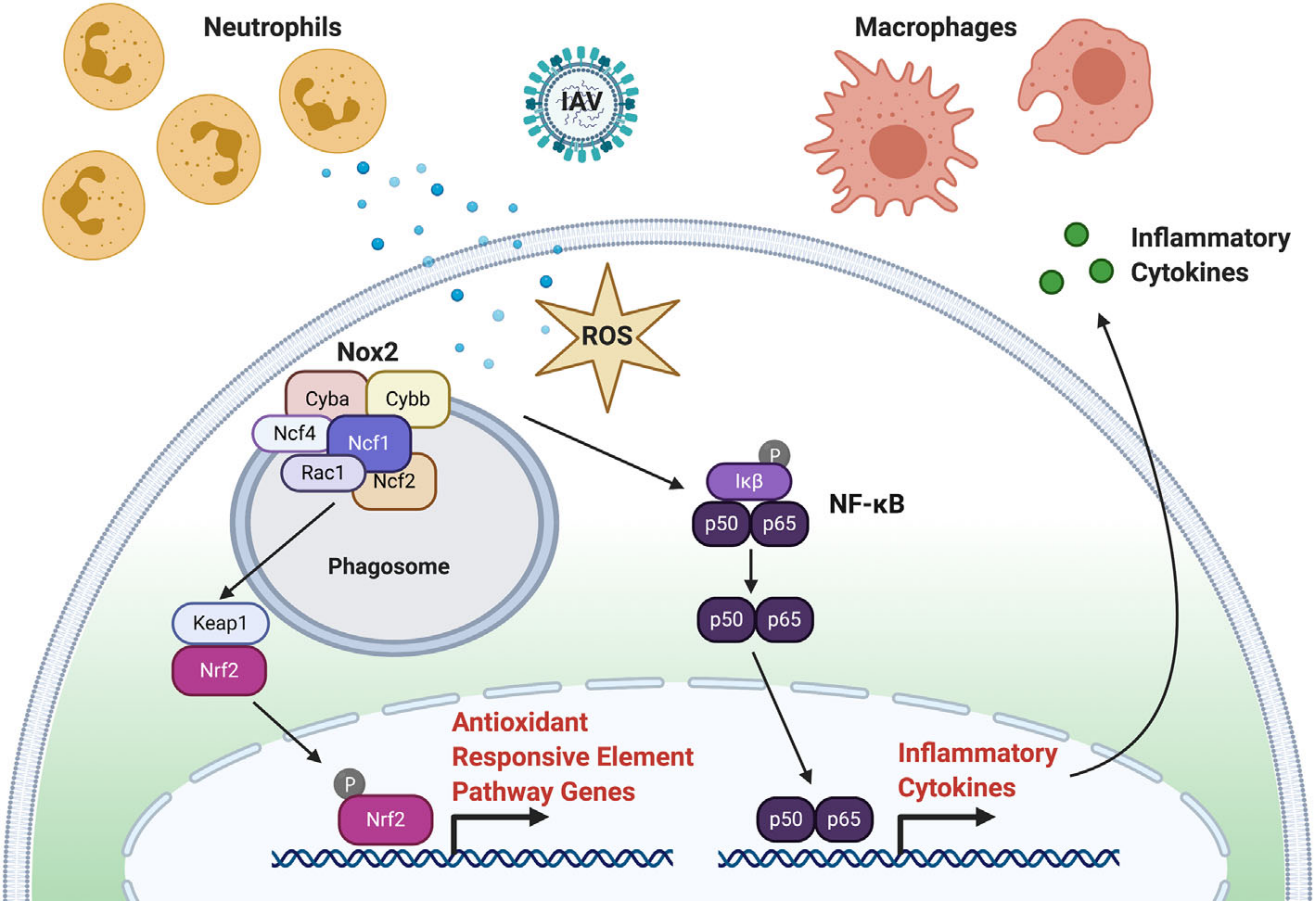
ROS Signaling in Response to Virus Infection. Following infection, production of ROS through the respiratory burst response function to recruit phagocytes (neutrophils and macrophages) to the site of infection and inactivate virus particles. Activation of the phagocyte nicotinamide adenine dinucleotide phosphate (NADPH) oxidase (PHOX) complex produces ROS. The PHOX complex is composed to Cyba, Cybb, Ncf1, Ncf2, Ncf4, and Rac1. Activated Nox2 can activate NFκB (p60, p65) that leads to subsequent inflammatory chemokine and cytokine expression. Activated Nox2 can also activate the NRF2 transcription factor through KEAP1 to initiate the expression of antioxidants.

One of the important functions of macrophages and neutrophils during infection and injury is a respiratory (also called oxidative) burst response that functions to recruit additional phagocytes and degrade pathogens. Following a respiratory burst response, reactive oxidative species (ROS), hydrogen peroxide (H_2_O_2_), and superoxide anion $O_{2}^{-}$ are produced by the phagocyte nicotinamide adenine dinucleotide phosphate (NADPH) oxidase (PHOX) complex (**Figure 2**). The PHOX complex is conserved between humans and zebrafish (119). The major catalytic component of PHOX, NOX2, is composed of p91^phox^ (encoded by *cybb*) and p22^phox^ (encoded by *cyba*) and is bound to the phagosome membrane. The activity of NOX2 is stabilized and activated by three regulatory subunits, p47^phox^ (encoded by *ncf1*), p67^phox^ (encoded by *ncf2*) and p40^phox^ (encoded by *ncf4*), along with the small GTPase, Rac (encoded by *rac1*). GTP-Rac interacts with NOX2 that, in turn, interacts with p67^phox^ to activate NOX2 at the phagosome membrane. P47^phox^ has major roles in both NOX2 activation and stabilization at the plasma membrane. First, phosphorylation of p47^phox^ exposes two SRC-homology 3 domains that interact with the proline-rich motif of the NOX2 subunit, p22^phox^. Second, additional PHOX homology domains on activated p47^phox^ can bind the phosphoinositide, phosphatidylinositol 3,4-bisphosphate (PI(3,4)P2), that is produced by phosphoinositide-3-OH kinase (PI(3)K). Activated PHOX produces superoxide through the reduction of oxygen into superoxide.

Humans with mutations in PHOX subunits may develop chronic granulomatous disease (CGD), which is characterized by inflammatory disorders, granuloma formation, and increased susceptibility to infection. Individuals with mutations p91^phox^ (*CYBB*), p22^phox^ (*CYBA*), p47^phox^ (*NCF1*), p67^phox^ (*NCF2*), or p40^phox^ (*NCF4*) develop CGD. Zebrafish have been used to model CGD in the context of fungal infection by *Aspergillus nidulans* (120). Zebrafish embryos with a mutation in p22^phox^ (*cyba^sa11798^*) were observed to have decreased survival to *A. nidulans* infection, similar to what has been observed in CGD patients with fungal infections. Neutrophil migration was disrupted in the homozygous mutants as recruitment that should have peaked at 24 h post-infection (hpi) continued to 96 hpi. Antisense morpholino knockdown of Ncf1 in zebrafish was shown to increase susceptibility to *Candida albicans* infection and decrease the respiratory burst response to infection (17, 18). In other studies PHOX has been inhibited using small molecules, such as diphenyleneiodonium (DPI) (121), VAS-2870, and Phox-I2 (122). DPI was shown to inhibit NOX and the production of superoxide generated by PMA (phorbol 12-myristate 13-acetate)-stimulated macrophages (121). VAS-2870 was first described to inhibit platelet growth factor (PDGF)-dependent NADPH ROS production in vascular smooth muscle cells (123), but has also been shown to inhibit NADPH oxidase activity in regulatory T cells to block the suppression of CD4+ cells (124). Phox-I2 was designed to target the Rac1 GTPase binding site on p67^phox^, and was shown to suppress ROS production in mouse neutrophils (122).

The NADPH oxidase (Nox) gene family in zebrafish is comprised of *nox1*, *cybb*, *nox4*, *nox5*, and the dual oxidases, *duox* and *duox2* (119). While Nox1 and Cybb are part of PHOX and regulated by cytosolic factors, Nox5, Duox and Duox2 are activated by calcium (Ca^2+^) as they share helix-loop-helix EF-hand domains. Like Cybb, Nox4 is stabilized by p22^phox^, but it is constitutively active. Nox family members also differ by their expression and roles in different tissues. For example, human *NOX1*, *NOX3*, *NOX4*, *NOX5*, and *DUOX2* are expressed in cardiovascular tissues. During the first 2 days of zebrafish embryonic development, the expression of *cybb* was stable during the first 2 days of development with *nox1*, *nox5* and *duox* being more dynamic (125). Zebrafish Duox was shown to be required for the recruitment of neutrophils to fin bud injury by generating a H_2_O_2_ gradient (26). Duox was also shown to be required for peripheral axon regeneration in zebrafish (126). Several NAPDH oxidase inhibitors have been developed in addition to DPI and VAS-2870, including the general NADPH inhibitor celastrol. Celastrol was shown to have higher inhibitory activity for Nox1 and Nox2 than Nox4 and Nox5 in zebrafish embryos (127). Nox1 inhibitors of human NOX1 include ML171 (128). GKT137831 and GKT136901 were shown to be an inhibitors of mouse NOX1 and NOX4 (129, 130). Specific NOX4 inhibitors include GLX7013114 (131), GKT137928 (132) and ACD084 (133). These and other small molecule inhibitors may be useful to screen for the relative contribution of different NADPH oxidases to inflammatory responses during viral infection.

The amount of ROS production following a respiratory burst response is indicative of the intensity of the immune response and overall health of the organism. A method to assay the respiratory burst response was developed for zebrafish embryos and adult tissues (134–136). This assay measures production of H_2_O_2_ in response to phorbol myristate acetate (PMA) by detecting the oxidation of dihydrodichlorofluorescein (H_2_DCF) to the fluorescent product, dichlorofluorescein (DCF) to determine the fold induction of the respiratory burst (16). These assays have been used to study how low-dose arsenic reduces the capacity of zebrafish embryos infected with SHRV to mount a respiratory burst response (137). The same assays have been used to measure the respiratory burst response in zebrafish embryos following bacterial (16, 137) and fungal infection (18). A single cell respiratory burst assay has been developed to complement “whole embryo” methods described above (138). Dissociated cells from zebrafish embryos are stimulated with an oxidant, such as rotenone or H_2_O_2_, incubated with a fluorescent ROS-detecting probe, such as CellROX, and then analyzed using FACS. ROS from specific cell types can be measured by assaying fluorescent reporter lines, such as neutrophils from the *Tg(mpx:EGFP)* line, to measure respiratory burst activity specifically in zebrafish neutrophils. This method has recently been used to study the roles of neutrophils in excessive inflammation following tissue injury in cystic fibrosis transmembrane conductance regulator (*cftr*) zebrafish mutants (139).

Apoptosis of neutrophils at the site of inflammation is one mechanism by which inflammation is resolved. A method to measure neutrophil apoptosis at the site of tailfin injury was developed for *Tg(mpx:GFP)^i114^* zebrafish embryos using immunohistochemistry to screen for pharmacological agents that could promote neutrophil apoptosis (140). Pyocyanin a phenazine pigment produced by *Pseudomonas aeruginosa*, and roscovitine, an inhibitor of cyclin-dependent kinases, both reduced the number of neutrophils at the site of injury at 24 hours post injury. Agents to delay neutrophil apoptosis and prolong inflammation were also screened. Of the agents tested, the dipeptide pan-caspase inhibitor, benzyloxycarbonyl-Val-Asp-fluoromethylketone (zVD.fmk), decreased neutrophil apoptosis the most. This inhibitor was previously shown to prolong inflammation following tailfin injury in zebrafish embyros (36).

The distribution of ROS in zebrafish embryos has been assayed using high resolution intravital imaging. ROS can be detected using fluorescent imaging of zebrafish embryos treated with the cell-permeable dye, dihydroethdium (DHE), that is sensitive to superoxide (141, 142). DHE has blue fluorescence until it is oxidized by superoxide to form oxyethidium that emits red fluorescence and intercalates with nucleic acids (143). Phan et al. developed a model of bacterial infection that stimulated neutrophil and macrophage activation by injecting *Escherichia coli* into the notocord that was impenetrable by phagocytes (144). The role of neutrophil generated superoxide to clear infection was characterized using this model. Using the DHE assay, superoxide production was observed in neutrophils of infected *Tg(mpx:GFP)* embryos compared to controls. The superoxide response was shown to be neutrophil specific by examining infected embryos treated with Lipo-Clodronate to deplete macrophages, and colony stimulating factor 3 receptor (Csf3r) morphants that had depleted neutrophils.

Intracellular hydrogen peroxide (H_2_O_2_) production has been visualized in zebrafish using the fluorescent reporter protein, HyPer (26, 145). H_2_O_2_ production following wounding in the tail bud of zebrafish larvae was visualized *in vivo* in the fluorescent reporter line, *Tg(actb:HyPer)*, that drives the expression of HyPer line using a β-actin (*actb*) promoter (26). This study demonstrated that a gradient of H_2_O_2_ after wounding was required for neutrophil recruitment to the site of injury. Visualization of H_2_O_2_ production within neutrophils after wounding was achieved using a zebrafish fluorescent reporter line, *Tg(lyz:HyPer)^ka4^*, that drives the expression of HyPer line using a *lyz* promoter (145).

### Additional Zebrafish Models to Study Neutrophil Function

Several additional zebrafish transgenic and mutant lines have been developed to study neutrophil function. Defects in neutrophil trafficking have been modeled using four different transgenic lines. Humans with Warts, Hypogammaglobulinemia, Infections, and Myelokathexis (WHIM) syndrome have mutations in the chemokine receptor, CXCR4. A zebrafish model of WHIM syndrome, *Tg1(-8mpx:cxcr4b-EGFP)^uwm3^*, was developed by expressing a truncated Cxcr4b protein tagged with a EGFP reporter in neutrophils using a *mpx* promoter (146). A dominant-negative *rac2* zebrafish line (*Tg(mpx:mCherry,rac2_D57N)^zf307^*) was used to show that Rac2 was required for neutrophil migration to a tailfin injury (147). As described in the non-coding RNA section of this review, the microRNAs, miR-722 (148, 149) and miR-199 (150), are two additional zebrafish neutrophil trafficking mutants. Defects in Mpx function have been modeled in the “spotless” mutant, *mpx^NL144^*, which has a premature stop codon in the *mpx* gene (151), and the *durif* mutant, *mpx^gl8/gl8^*, which has cis-acting point mutation in *mpx* (145). Myeloperoxidase activity was absent in these mutants, as assayed using Mpx TSA and anti-nitrotyrosine staining (151). These models are complementary to Csf3r morphants that have depleted neutrophils (144, 152).

## Zebrafish Models of Viral Infection

The zebrafish is a powerful model system for the study of virus infection and host immune response. Initial studies involved using the zebrafish to model fish viruses to develop strategies for mitigation, including fish virus vaccines. These studies often focused on critical factors like temperature and route of infection (immersion and different forms of injection) in order to replicate viral disease observed in other fish species. With time came the recognition that zebrafish viral infection models could also be used to study the host immune responses. These studies have become more sophisticated, moving from the realm of pathology and interferon and interferon-stimulated genes responses to more complex studies examining issues such as immune cell behavior. The zebrafish is uniquely positioned as a model in this regard due to the generation of various transgenic lines that label immune cells such as neutrophils and macrophages. As discussed previously, zebrafish possess numerous inherent advantages that make this type of investigation possible, including near transparency during the embryonic and larval periods of development, an array of forward and reverse genetics tools, and deeply sequenced genome. These advantages enable directed studies at the host-viral pathogen interface, where it is possible to answer questions about how cells like macrophages and neutrophils work to limit the spread of infection and regulate the inflammatory rheostat. Below is a summary of several viral models that have been developed in zebrafish, including fish viruses, human viruses that infect zebrafish, and xenograft models. Additional information about these and other viruses can be found in **Table 5**.

**Table 5:** Viruses studied in zebrafish.

Virus Family	Virus	Preferred Host	Method(s) of Infection	Zebrafish or Human Receptor
**Group I: Double-stranded DNA Viruses**				
*Herpesviridae*	Cytomegalovirus (CMV)	Human	One-cell stage injection with pUL97 plasmid (153)	Human: OR14l1 (154)
	Herpes simplex virus type 1 (HSV-1)	Human	Inoculation by injection in the dorsal telencephalon or olfactory bulb (155–159)	Zebrafish: Hs3st4 (156) Human: HS3ST4
	Kaposi’s sarcoma-associated herpesvirus (KSHV or HHV8)	Human	Xenograft (160)	Zebrafish^#^: Cd209, Itga3b, Itga5 Human: heparin sulfate, CD209, ITGA3, ITGA5
*Iridoviridae*	European sheatfish virus (ESV)	Fish	Immersion (161)	Unknown
	Infectious spleen and kidney necrosis virus (ISKNV)	Fish	Intraperitoneal injection, natural occurrence (162–164)	Unknown
	Lymphocystis disease virus (LCDV)	Fish	Intraperitoneal injection (165)	Unknown
**Group III: Double-stranded RNA Viruses**				
*Birnaviridae*	Infectious pancreatic necrosis virus (IPNV)	Fish	Vertical transfer (female), natural occurrence, immersion, intraperitoneal injection (61, 166, 167)	Unknown
**Group IV: Positive Sense Single-stranded RNA Viruses**				
*Nodaviridae*	Betanodavirus (nervous necrosis virus) (NNV)	Fish	Intraperitoneal injection, natural occurrence, immersion (168–171)	Zebrafish^#^: Hspa8
*Caliciviridae*	Norovirus (NoV)	Human	Yolk sac injection, immersion (172)	Unknown
*Picornaviridae*	Cyprivirus	Zebrafish	Natural occurrence (173)	Unknown
*Togaviridae*	Chikungunya virus (CHIKV)	Mosquito, Human	Caudal vein, aorta (94, 117, 174)	Unknown
	Sindbis virus	Mosquito, Birds	Caudal vein, aorta (175, 176)	Zebrafish^#^: rpsa Human: heparin sulfate, RPSA
*Flaviviridae*	Zika virus	Mosquito, Human	Xenograft (177)	Zebrafish^#^: Axl Human: AXL (178)
*Retroviridae*	Zebrafish endogenous retrovirus (ZFERV)	Fish	Natural occurrence (179, 180)	Unknown
**Group V: Negative Sense Single-stranded RNA Viruses**				
*Rhabdoviridae*	Spring viraemia of carp virus (SVCV)	Fish	Immersion, intraperitoneal injection, duct of Cuvier (51, 57, 61, 108, 181–183)	Unknown
	Snakehead rhabdovirus (SHRV)	Fish	Immersion, intraperitoneal injection (59, 60, 65, 184)	Unknown
	Piscine novirhabdovirus (VHSV)	Fish	Immersion, intraperitoneal injection (61, 185–188)	Unknown
	Infectious hematopoietic necrosis virus (IHNV)	Fish	Intraperitoneal injection, immersion, caudal vein, aorta (61, 108, 117, 166, 189–191)	Unknown
*Orthomyxoviridae*	Influenza A virus (IAV)	Human	Duct of Cuvier, swimbladder (8, 24, 25)	Zebrafish: Sialic acid (8) Human: Sialic acid
*Flaviviridae*	Dengue virus (DENV)	Mosquito, Human	Intraperitoneal injection (192)	Zebrafish^#^: Cd209, Rab5aa, Rab5ab, Hspa5 Human: CD209, RAB5A, HSPA5,
*Amnoonviridae*	Tilapia lake virus (TiLV)	Fish	Immersion, intraperitoneal injection (53)	Unknown
**Group VII: Double-stranded DNA Viruses With an RNA Intermediate in Their Life Cycle**				
*Hepadnaviridae*	Hepatitis b virus (HBV)	Human	One-cell stage injection with transgenic plasmid (193–195)	Zebrafish^#^: Slc10a1 Human: SLC10A1
	Hepatitis c virus (HCV)	Human	One-cell stage injection with transgenic plasmid (194, 196, 197)	Zebrafish^#^: Cd81a, Cd81b, Scarb1, Cldn1, Oclna, Oclnb, Npc1l1 Human: CD81, SCARB1, CLDN1, OCLN, NPC1L1

^#^Zebrafish ortholog identified using the Zebrafish Information Resource (ZFIN; https://zfin.org).

### Fish Viruses for Heterologous Gene Expression

Some of the earliest published virus studies performed in zebrafish used vesicular stomatitis virus (VSV) envelope containing glycoprotein (VSVG) pseudo-typed retroviruses. These efforts demonstrated that it was possible to stably transfer and express genes in zebrafish *via* retroviral vectors (198–200), albeit at efficiencies lower than seen in human cells. Subsequently, the fish rhabdovirus IHNV [also formerly known as Oncorhynchus 1 novirhabdovirus now preferably known as the salmonid novirhabdovirus (201, 202)] and the aquatic birnavirus infectious pancreatic necrosis virus (IPNV) was shown to trigger infections in adult zebrafish following intraperitoneal injection and improve viral infection efficiency (166). In this study, it was noted that the infections particularly affected the head kidney, the principal site of hematopoiesis in the fishes, and that hematopoietic cells were affected. The results supported a role for this approach in complementing VSVG heterologous gene expression studies.

### Fish Viruses

#### Spring Viremia of Carp Virus (SVCV)

The spring viremia of carp virus (SVCV), a species of virus belonging to the genus *Vesiculovirus* of the *Rhabdoviridae* family, is associated with acute infectious dropsy of carp and spring viremia of carp (181). Naturally occurring infections have been detected in numerous cyprinid species, and SVCV has been isolated from Nile tilapia and rainbow trout (203, 204). To better understand the disease process, a model in which adult zebrafish were challenged with SVCV by immersion was developed to mimic a natural route of infection (181). Zebrafish are typically maintained at 28°C-28.5°C to mimic their natural environment. Lethal SVCV infections most often occur at temperatures below 15°C. In order to more closely model a natural infection, zebrafish were acclimated to lower temperatures and exposed by immersion to differing doses of SVCV. Several profound gross pathological changes that resembled natural infections were noted in zebrafish exposed to these lower temperatures; however, many of the histological changes that are typically noted in natural infections (e.g. edema, hemorrhage, inflammation, and necrosis) were not observed. This was attributed to the fact that the zebrafish were not able to mount a robust immune response at 15°C or 20°C as their natural environment is approximately 28°C.

Another larval zebrafish model for SVCV infection was developed in which virus was injected into the systemic circulation *via* the caudal vein (57). Using this model, several ISGs were induced following SVCV infection, including *rsad2*, *mxa*, and *mxb*. Levraud et al (57) further adapted their SVCV model by introducing a morpholino-mediated, loss-of-function approach that knocked down Ifnphi1 expression. Survival to SVCV infection was improved in transgenic embryos that overexpressed *ifnphi1* using beta-actin promoter. In addition, they identified Crfb1 and Crfb5 as subunits of the zebrafish IFN receptor complex, as Crfb1 and Crfb5 morphants lacked an interferon antiviral response to SVCV infection.

Lopez-Munoz et al. (182) developed an immersion model for SVCV infection using zebrafish larvae. They observed that 3 dpf larvae exposed to SVCV at 26°C were susceptible to infection, with 50% survival seen between 3- and 4-days post-infection (dpi). In addition, using their immersion strategy, they observed that SVCV failed to induce a robust antiviral IFN response, although there was evidence of a strong pro-inflammatory response with increased *il1b*, *tnfa*, and *lta* expression. Espín-Palazón et al. (55) applied a larval SVCV immersion model to determine that the pleiotropic pro-inflammatory cytokine Tnfa functioned to inhibit SVCV clearance by blocking autophagy in the host. Using the LC3-GFP autophagy transgenic line [*Tg(CMV : EGFP-map1lc3b)*] (205) and the zebrafish ZF4 fibroblast cell line, the authors found that Tnfa inhibits the formation of autophagosomes during viral infections. Libran-Perez et al. (206) further investigated the importance of autophagy in SVCV infection using the zebrafish larval infection model. They determined that exposure to palmitic acid, an anti-inflammatory compound known to induce autophagy, could increase zebrafish survival and reduce viral load and replication.

There have been three studies aimed at understanding the effects of SVCV infection on the transcriptomes of adult zebrafish (118, 183, 207). Encinas et al. (183) performed a microarray study in an effort to identify genes that participate in multiple pathways in the antiviral response and upon survival and were significantly up-regulated or down-regulated. They argued that specific targeting of these genes with candidate drugs could be an effective strategy in mitigating impacts on fisheries of SVCV. Wang et al. (118) performed a high-throughput RNA sequencing (RNA-Seq) experiment using brain and spleen tissue derived from SVCV-infected and control adult zebrafish. They identified 382 differentially expressed genes in the brain and 926 differentially expressed genes in the spleen. In each study, the authors identified differential expression of genes associated with inflammation and immunity. Valenzuela-Muñoz et al. (207) performed an RNA-Seq experiment comparing the long non-coding RNA (lncRNA) transcriptomes of kidney tissue from control and *rag^+/-^* heterozygous adult zebrafish following SVCV infection. As described later in this review, putative functional annotation of candidate lncRNA were assigned using Gene Ontology (GO) terms annotated to protein-coding genes within the proximity of the lncRNA (10 kbp up- or down-stream). Using this approach, the authors identified lncRNA genes associated with adaptive immunity based on their differential expression in the *rag1^+/-^* heterozygotes. In addition, they also identified lncRNA genes that could be linked to metabolic processes, including the activation of immune cells, and to positive regulation of TOR signaling, which may lead to the inhibition of autophagy. The authors noted that autophagy has been linked to both pro-viral and anti-viral responses.

#### Infectious Spleen and Kidney Necrosis Virus (ISKNV)

The infectious spleen and kidney necrosis virus (ISKNV) belongs to the genus *Megalocytivirus* in the family *Iridoviridae*. ISKNV and ISKNV-like viruses infect more than 50 marine fish species and impact fisheries of commercial value (208). In fact, natural infections of laboratory zebrafish have been noted (162). These zebrafish infections exhibited bloating, elevation of scales, and petechial hemorrhaging in adults. Xu et al. (163) developed an ISKNV adult zebrafish infection model using intraperitoneal injections of virus. Zebrafish infected with ISKNV exhibited mortalities and clinical symptoms reminiscent of natural infections, including elevation of scales and petechia. In addition, the virus induced cellular hypertrophy in the kidney and spleen. In a follow-up study comparing the course of ISKNV infection in *Tetraodon nigroviridis* and zebrafish, Xu et al. (209) showed significant induction of *ifnphi1* and *tnfa* transcription in zebrafish, which is indicative of robust antiviral and pro-inflammatory responses to infection.

#### Piscine novirhabdovirus (Formerly Oncorhynchus 2 Novirhabdovirus or Viral Hemorrhagic Septicemia Virus [VHSV] or Egtved Virus)

Piscine novirhabdovirus belongs to the *Novirhabdovirus* genus of the *Rhabdoviridae* family and causes a prolific viral disease that afflicts over 50 freshwater and marine species in the northern hemisphere (201, 202). Novoa et al. (185) developed juvenile and adult zebrafish immersion and intraperitoneal injection models for piscine novirhabdovirus infection. They observed that adult zebrafish infected by intraperitoneal injection developed disease similar to that found in nature, with evidence of petechial hemorrhage, exophthalmoses, distended visceral cavities, and erratic swimming behaviors. Further, they observed in the kidney increased expression of gene transcripts associated with antiviral and pro-inflammatory responses, including *tlr3, ifnphi1, mxa, ifng1*, and *tnfa*. Novoa et al. (185) also demonstrated that a recombinant salmonid novirhabdovirus (IHNV) lacking an NV gene, but expressing piscine novirhabdovirus G gene, had dose-dependent protective effects for zebrafish in resisting piscine novirhabdovirus infection, as measured by a significant reduction mortality.

#### Snakehead Rhabdovirus (SHRV)

Snakehead rhabdovirus (SHRV) belongs to the *Novirhabdovirus* genera of the family *Rhabdoviridae* and is closely related to the other commercially significant viruses IHNV and VHSV. We have previously published a comprehensive characterization of SHRV infection in zebrafish (59). Our laboratory group developed and applied embryonic and adult zebrafish models for SHRV infection to address questions related to the host immune and inflammatory response to infection (59, 60, 65). Zebrafish between 24 hpf and 30 dpf were susceptible to infection by immersion, while adult zebrafish could only be infected by intraperitoneal injection. Infected zebrafish presented with petechia, abdominal redness, and erratic swim behaviors. Histological examination of embryonic and juvenile fish revealed evidence of inflammation, including pharyngeal epithelium and liver necrosis and congestion of the swim bladder by cellular debris. There was also evidence of monocyte accumulation in the infected areas, which is indicative of inflammation. Adult fish infected with SHRV exhibited more localized effects closer to the site of infection, including evidence of inflammation with edema, petechia, and fluid and immune cell accumulation in the abdomen. In addition, SHRV infection by immersion induced expression of antiviral *ifnphi1* and *mxa* transcripts. In another study, Phelan et al. (65) determined that SHRV upregulated expression of the immune genes *traf6* and *tlr3* and slightly downregulated the expression of *irak4* in both embryonic and adult zebrafish. Gabor et al. (60) showed that the overexpression of a full-length Mda5 was protective against SHRV infection, while overexpression of a dominant-negative Mda5 receptor (with a CARD domain deletion) could increase SHRV mortality. Kortum et al. (184) applied the adult SHRV infection model to characterize its effects on polymeric immunoglobulin (Ig) receptor (pIgR) expression. pIgR expression is thought to be regulated by Tlr3 and Tlr4 signaling and to link aspects of the innate immune response to the adaptive immune response (210). Upon SHRV infection, Kortum et al. (184) observed that *pigr* and *pigrl* transcripts were reduced, leading to speculation that SHRV suppresses the immune response, at least in part, through this mechanism.

#### Zebrafish Picornavirus-1 (ZfPV-1)

Recently, evidence for a natural picornavirus infection in the zebrafish gut was detected in a viral metagenomics analysis of zebrafish gut tissue (173). *In situ* hybridization revealed infection of the apical surfaces of enterocytes, as well as near the mucosal layer and within the lumen of the intestine. While AB zebrafish infected with ZfPV-1 were asymptomatic, the virus appears to be widespread in research facilities, with 56% of the 41 institutions tested exhibiting evidence of infection within the fish populations. The prevalence of ZfPV-1 in wild populations has not been determined. Development of a picornavirus model that can infect zebrafish naturally and not trigger symptoms has the potential to reveal novel insights into the underpinnings of the host-pathogen interaction in a low-level infection. It may be possible to gain an understanding of the role these viruses play in dysregulating immune and inflammatory responses over time, including in the presence of secondary infections, and in affecting embryonic development. In addition, a zebrafish picornavirus model could be applied to test the immune robustness of different zebrafish strains as well as the importance of various immune responsive genes.

As described, there are numerous advantages to modeling fish viruses in the zebrafish. The ability to have an easily maintained, relatively low cost, teleost model to study viral infection makes it possible to study an array of research questions. There are several challenges that need to be overcome in order to model viral disease, including determining the appropriate life stage, potential issues with viral tropism, and especially difficult hurdles related to temperature. Nevertheless, there is now a considerable body of literature demonstrating the usefulness of the zebrafish models in the study of fish viruses and immune response. It is particularly noteworthy that many of these viruses can be modeled during the embryonic and larval periods. This ability to infect embryonic and larval fish enables researchers to ask far more precise questions, particularly in the realm of host-virus interaction and immune response. Future studies should take advantage of these developing models to answer critical questions related to vertebrate immune responses to viruses that are universal and conserved across all species.

### Human and Mammalian Viruses

Zebrafish possess many of the same receptors required by human and other mammalian viruses for entry and infection (**Table 5**). The following summarizes some of the human virus research that has been conducted in the zebrafish model. These studies highlight the flexibility of the zebrafish model, particularly with regard to its ability to acclimate and then be infected by viruses that are typically most virulent in temperature ranges more conducive to humans and mammals.

#### Chikungunya Virus

Chikungunya virus (CHIKV) is a single-stranded, positive-sense *Alphavirus* that causes acute, febrile illnesses accompanied by severe arthralgia (211). CHIKV is a mosquito-borne virus endemic to Africa, Asia and the Indian subcontinent, although there have been outbreaks in other parts of the world, including in the regions of the Americas (212). Palha et al. (94) developed a larval zebrafish model for CHIKV infection. Using a GFP-labeled CHIKV, the authors observed the development of a systemic infection that largely resolved by 4 days post-infection (dpi). Interestingly, CHIKV infections persisted in the brain parenchyma until at least 7 dpi. CHIKV induced a powerful type I interferon response, as measured by *ifnphi1* expression, that was largely mediated by neutrophils and hepatocytes. The role neutrophils played in producing this antiviral *ifnphi1* response was particularly intriguing because their function in viral infections has not been fully appreciated. These findings were bolstered by experiments that compared the relative importance of macrophages and neutrophils in containing CHIKV infections. Palha et al. (94) observed that reductions in neutrophil populations (induced by morpholino knockdown of Csf3r) made zebrafish more susceptible to CHIKV infection, while macrophage depletion by a drug-inducible cell ablation system led to only a modest increase in disease severity.

Briolat et al. (117) performed microarrays on larval zebrafish that had been infected with either IHNV or CHIKV. Each of these viruses has different disease kinetics and induce differing type I interferon response. While IHNV stimulates a milder type I interferon response, CHIKV induces a far more robust expression. Using the microarray approach, the authors identified a suite of zebrafish ISGs that they could compare to human studies. With this information, Briolat et al. (117) identified ISGs that are conserved across vertebrate species.

#### Sindbis Virus

Like CHIKV, the Sindbis Virus (SINV) is an *Alphavirus* capable of neuroinvasion. Passoni et al. (175) developed a larval SINV infection model in the zebrafish and observed that the virus could infect multiple organs and replicate throughout the larvae. Further, they established the means by which CHIKV and SINV entered the central nervous system. Based on the data they collected, Passoni et al. (175) speculated that CHIKV enters the CNS by infecting the brain microvasculature endothelial cells at the blood-brain barrier and that SINV enters the CNS through axonal transport *via* the peripheral nerves.

Boucontet et al. (176) observed that larval zebrafish infected with SINV exhibited increased mortality when infected secondarily with the bacterium *Shigella flexneri*. The authors also noted increased bacterial burdens in those animals that were infected with SINV first and *S. flexneri* second. The initial viral infection induced expression of antiviral *ifnphi1*, pro-inflammatory *tnfa* and *il1b*, and anti-inflammatory *Il10* transcripts. It also affected neutrophil populations, function, and behavior. Specifically, Boucontet et al. (176) noted fewer neutrophils and more dying neutrophils in larvae that had been infected with SINV and then *S. flexneri*. Interestingly, they noted an increase in neutrophils by 120 hpi when zebrafish were infected with SINV. The authors speculated that the SINV infection triggers an IFN polarization that renders affected cell populations unable to mount antibacterial responses. They also observed the neutrophils exhibited defects in recruitment to areas of infection, and they attributed this finding to the upregulation of *il10* that was observed. Taken together, these data indicate an important role for neutrophils in containing secondary infections following SINV infections and offer this superinfection model as a means to test these phenomena.

#### Dengue Virus

Dengue virus (DENV) is a single-stranded, positive-sense, mosquito-borne *Flavivirus* that can induce a broad range of manifestations in infected humans, from asymptomatic to severe flu-like. Recently, Balkrishna et al. (192) described an adult zebrafish model for Dengue virus serotype 3 (DENV-3) infection. The authors collected serum containing DENV-3 from human subjects and then performed intramuscular injections of serum into adult zebrafish that served as carriers to propagate the virus. After 14 days, serum from infected zebrafish was harvested, diluted, and injected intramuscularly into secondary adult zebrafish, which served as the study subjects. Using a qPCR-based approach to measure DENV-3-specific transcripts, Balkrishna et al. (192) observed a viral load that was sustained through 15 days post-injection. Histological analysis of the liver indicated necrosis, increased numbers of inflammatory cells, and increased presence of erythrocytes. Blood smears indicated increasing numbers of leukocytes over the course of infection, decreasing numbers of erythrocytes, and decreased numbers of platelets, which is commonly seen in human DENV infections. Close inspection of caudal fins revealed evidence for DENV-induced hemorrhage that was not seen in control groups. Further, increases in the expression of *ang2*, a pro-angiogenic gene and indicator of inflammation, and *ccl3*, a chemokine, were noted. The ayurvedic herbal drug, Denguenil, was shown to limit the effects of DENV-3 infection in this zebrafish model in a dose-dependent manner, as evidenced by decreased levels of necrosis, reduced numbers of inflammatory cells, and decreased levels of erythrocytes in the liver; decreased number of leukocytes, increased numbers of erythrocytes, and decreased numbers of platelets in blood smears; diminished evidence of hemorrhage in caudal fins; and decreases in the levels of *ang2* and *ccl3* transcripts.

#### Human Noroviruses

Human noroviruses are single-stranded, positive-sense, non-enveloped RNA viruses belonging to the family *Caliciviridae* and are the primary causes of viral gastroenteritis. Van Dycke et al. (172) recently described a larval zebrafish model for human norovirus infection. Zebrafish at 3 d post-fertilization were subjected to yolk injections of human norovirus collected from the stool of human test subjects. A concurrent set of experiments with mouse norovirus was conducted, but it was determined the mouse noroviruses could not cause infections. The authors observed that human norovirus replicated in zebrafish, as detected by qPCR assays designed to detect viral RNA copies. These data were supported by ELISA, in which evidence of increased viral antigens was observed. Human norovirus replication was detected by immunohistochemistry in both the intestine and caudal hematopoietic tissue of the larval zebrafish. These findings supported the idea that there is a dual tropism for human noroviruses in zebrafish. Infections with human norovirus also induced antiviral responses in the zebrafish, as evidenced by significant increases in the expression of *ifnphi1*, *mxa*, and *rsad2* transcripts relative to controls. Zebrafish infected with the human norovirus exhibited significant reductions in viral load following exposure by immersion to the antiviral compound 2’-C-methylcytidine (2CMC) (as measured by EIA). These findings demonstrated the utility of this infection model for testing antiviral drugs.

#### Herpes Simplex Virus – Type 1

Herpes simplex virus – type 1 (HSV-1) is a double-stranded DNA virus that belongs to the *Alphaherpesviridae* subfamily. In humans, HSV-1 may be transmitted by saliva or other bodily secretions. It is most often associated with cold sores, but can also cause an array of other herpetic lesions, including herpetic sycosis, herpes gladiatorum, and herpetic whitlow (213). Burgos et al. (155) developed an adult zebrafish model for HSV-1 infection. Following intraperitoneal injections, zebrafish were monitored for the presence of HSV-1 DNA. Between 1- and 4-days post-infection, zebrafish experienced active infection, as demonstrated by the presence of HSV-1 DNA. In addition, histological examination of zebrafish injected with HSV-1 demonstrated that there was a concomitant inflammatory response, even at sites distal to the site of injection. There were indications of degeneration of secondary oocytes and hemorrhage within the muscle tissue. The authors also noted tropism for neuronal tissue by the HSV-1.

Human heparan sulfate modifying enzyme 3-O-sulfotransferase-3 (3-OST-3) functions as a cellular receptor for HSV-1 infection. Zebrafish express multiple isoforms of (3-OST) (214). Several studies were performed in which the zebrafish 3-OST isoforms 3-OST-2, 3-OST-3, and 3-OST-4 were heterologously expressed in hamster CHO-K1 cells. CHO-K1 cells are normally resistant to HSV-1 infection; however, when the zebrafish 3-OST isoforms 3-OST-2, 3-OST-3, and 3-OST-4, and 3-OST-6 were heterologously expressed, these cells became sensitive to HSV-1 infection (156, 215–217). Interestingly, both zebrafish 3-OST-2 and 3-OST-4 are widely expressed in the central nervous system. Because of this, zebrafish may represent an ideal model in which to study effects of HSV-1 infection on the central nervous system and test potential therapeutics (156).

Ge et al. (157) demonstrated that HSV-1 could infect zebrafish at different larval stages from 48-96 hpf. They noted that HSV-1 infection triggered potent antiviral responses that included the upregulation of IFN and ISGs, including *isg15* and *rsad2*. While they demonstrated that the antiviral response that was generated was mediated through a Sting1-mediated cytosolic DNA sensing pathway initiated by Dhx9 and Ddx41 orthologues, they surprisingly found that cyclic GMP-AMP synthase (*cgas*) was not required for Sting1 signaling. These data support a mechanism by which zebrafish can mount a robust Sting-mediated inflammatory response, as has been demonstrated in other models (218).

#### Hepatitis Viruses

Similar to DENV, hepatitis C virus (HCV) is a single-stranded, positive-sense RNA virus belonging to the *Flaviviridae* family of viruses. In addition to causing hepatitis, or inflammation of the liver, persistent HCV infections can lead to hepatocellular cancer. To date, no vaccine has been developed to prevent HCV infection. *In vitro* HCV studies had proven difficult until the development of subgenomic replicons that replicate autonomously (12, 219). Ding et al. (196) recently adapted a subgenomic replication scheme for use in zebrafish to model HCV replication in a live animal. In their study, the authors demonstrated by the presence of HCV transcripts that replication occurred. In addition, they observed that HCV replication could be inhibited by the drugs ribavirin and oxymatrine. Ding et al. (196) also noted expression of the HCV subgenome transcripts in the zebrafish liver and that this disrupted the expression of homologous genes similarly affected in human HCV-infected liver cells. These data indicated that this zebrafish model effectively recapitulates aspects of HCV infection and may be useful in better understanding the effects of HCV-triggered inflammation on transformation to hepatocellular cancer

Li et al. (220) modified this HCV model to restrict its expression to the zebrafish liver. Using this zebrafish liver-specific HCV subgenomic replication model, the authors observed opposing effects on autophagy when either human ATG10 or ATG10S was overexpressed. Specifically, ATG10 overexpression triggered amplification of the HCV-subgenomic replicons, while ATG10S overexpression caused their degradation. These data, coupled with data from experiments using the autophagy inhibitors 3MA and CQ, provide evidence for how autophagy may influence aspects of HCV replication. Because of the linkages between autophagy and inflammation (221), this model may facilitate studies aimed at understanding these processes in the context of HCV.

#### Influenza A Virus (IAV)

We have described zebrafish models for IAV infection that resemble human disease (8, 24). We demonstrated that zebrafish possess the α-2,6-linked sialic acid residues on their cells that provide IAV viruses a way to bind, attach, and enter cells. We showed that two different strains of IAV (A/PR/8/34 [H1N] and X-31 A/Aichi/68 [H3N2]) could infect, replicate, and cause mortality when injected into the circulatory system of a larval zebrafish. Using a recombinant IAV strain carrying a GFP reporter (NS1-GFP) (222), we demonstrated the progression of an infection that could be monitored by fluorescence microscopy. In addition to being a model for disseminated infection, we also developed a model for localized IAV infection using the swimbladder (8, 24), which is considered the functional analogue of the human lung in fish (223). Zebrafish infected with IAV produce strong antiviral responses, as measured by increased expression of *ifnphi1* and *mxa*. Zebrafish also exhibit strong pro-inflammatory responses to IAV infection, with increases in the expression of *il1b* and *cxcl8* transcripts observed, increased NFκB activation as noted in *Tg(6xHsa.NFκB : EGFP)* transgenic fish, and extensive damage to zebrafish muscle fibers, with neutrophils recruited to sites proximal to the unanchored ends of some fibers (25).

#### Zika Virus (ZIKV)

The Zika virus (ZIKV) is a positive sense, single-stranded, enveloped RNA virus belonging to the *Flaviviridae* family (224). ZIKV is transmitted to humans primarily by some types of *Aedes* mosquitoes (*A. aegypti* and *A. albopictus*), but there are other modes of transmission, including through sexual intercourse, laboratory exposure, blood transfusion, and from mother to fetus during the pre- and peri-natal periods. Most ZIKV infections trigger mild symptoms, including rash, fever, joint pain, and/or non-purulent conjunctivitis; however, ZIKV infections during pregnancy can have profound effects on the developing fetus’ nervous system. These may include congenital Zika syndrome (CZS), which is characterized by severe microcephaly accompanying the fetal brain disruption sequence (FBDS), as well as other brain and ocular defects and congenital contractures (225).

Ayala-Nunez et al. (177) developed a xenotypic system in their study aimed at understanding the role infected human monocytes play in disseminating ZIKV to the neural cells. In their model, they labeled human CD14+ monocytes with the dye CellTrace Yellow and injected them *via* the duct of Cuvier into the circulation of 48 hpf zebrafish embryos. By performing live imaging, the authors observed that monocytes infected with ZIKV exhibited increased capacity for transmigration. They also noted that monocytes exposed to ZIKV were more prone to arrest in zebrafish vessels and suggested that this behavior may facilitate attachment to the endothelial cells of the blood vessel. These data support a likely role for the microenvironment in mediating transmigration. We speculate that this zebrafish model could be applied to study the effects an inflammatory microenvironment has on monocyte transmigration when infected with ZIKV. It is worthwhile to note that ZIKV infects human cells that are cultured at temperatures 10°C higher than zebrafish embryos and the cooler temperature may alter function of the ZIKV-infected human monocytes. A follow-up experiment in the same study was performed using a transwell migration assay system in which infected human monocytes were added to a well containing a porous membrane layered with cells mimicking the blood brain barrier. Under the transwell, neural organoids were added. In this experiment, more ZIKV-infected monocytes were observed to migrate across the membrane than control monocytes. Further, the authors noted infection of the neural organoids by ZIKV, which indicated viral dissemination by the monocytes.

These research studies, coupled with the studies conducted with fish viruses, demonstrate the strength of the zebrafish model and highlight several of its attributes, including its fully sequenced genome, which allows for the identification of putative viral receptors that can often be inferred based on homology. The zebrafish model has been aided by the development of a variety of fluorescent reporter lines that label immune and other cells. Regarding host-virus interactions, there has been a wealth of knowledge garnered through the development of alternative vertebrate model systems. Nevertheless, the zebrafish model allows researchers to investigate questions often more difficult to answer in these other models. As an example, alternative vertebrate models for influenza A infection exist, including those in mice, guinea pigs, cotton rats, hamsters, ferrets, and macaques (226, 227). Each have distinct advantages and disadvantages, but none is ideal. For example, the mouse model is limited by the fact that many human influenza A viruses are unable to infect it due to differences in the viral receptors they possess. On the other hand, the ferret model possesses similar viral receptors to humans and mimics the viral kinetics most closely, but it is difficult to use due to its relative size and cost of husbandry, in addition to a lack of reagents and methods. When applied to appropriate research questions, zebrafish can have real advantages over mice, ferrets, and these other vertebrate models, particularly in areas related to neutrophil and macrophage biology. Using the zebrafish model, it is possible to track individual cells and ascertain their role in host defense and host inflammation using the full array of transgenic reporter lines and other reagents available. As described above, in each of the other human viruses tested, the zebrafish model has been utilized to make significant contributions. It is important for researchers interested in modeling virus infections to recognize the strengths and limitations of their respective models. Cross-model approaches have the potential to illuminate areas of host-virus biology that cannot be observed otherwise.

## Neutrophils and Hyperinflammatory Tissue Damage

Tissue damage can be caused by neutrophils when they fail to properly resolve inflammation. This can occur when neutrophils become over-activated and/or the number of neutrophils at the site are not reduced. In this uncontrolled response, neutrophils and macrophages recruited by these cytokines can destabilize the vasculature and damage tissues as they migrate to the site of infection as shown in a mouse model of IAV infection (228). Reduction of the number of neutrophils at a site of inflammation can occur by pyroptosis and reverse transendothelial migration. Failure to reduce the number of neutrophils at the site of inflammation can result in tissue damage (229). The inflammatory cytokine, *il1b*, has been shown to have a critical role in prolonged inflammation in the zebrafish notochord that cannot be infiltrated by macrophages and neutrophils during early stages of bacterial infection (230). Knockdown of Il1b was used to demonstrate that Il1b was required for the recruitment of neutrophils to the notochord. The same study also described how neutrophils can degranulate without having direct interaction with a pathogen. A subsequent study identified how neutrophil-generated ROS cleared bacterial infection of the notochord even though neutrophils cannot infiltrate the notochord (144).

Damage to skeletal muscle was observed in a zebrafish model of IAV infection (25). By 24 hours post infection, zebrafish embryos were observed to have mild muscle degeneration with sarcolemma damage and defects in extracellular matrix adhesion. Confocal imaging of IAV-infected *Tg(mpx:EGFP)* showed that neutrophils localized to sites of fiber damage. Muscular degeneration phenotypes observed in the zebrafish model of Duchenne Muscular Dystrophy, *dmd^ta222a/ta222a^*, were found to be exacerbated following IAV infection.

## Transcriptional Profiling to Identify Stages of Hyperinflammatory Response

High-throughput RNA sequencing (RNA-Seq) of bulk tissues has begun to be applied to study zebrafish models of viral infection. The response to SVCV infection in zebrafish was characterized by RNA-Seq in the brain and spleen tissues (118). Levraud et al. (174) used RNA-Seq to characterize the response to CHIKV infection following morpholino-mediated knockdown of Crfb1 and Crfb2. Another important aspect of this study was identifying 97 ISGs that had human orthologs previously identified as ISGs in other studies. Another study of SVCV infection profiled gene expression in kidneys at 24 hpi in six-month old adult zebrafish with and without an impaired adaptive immune system by comparing heterozygous *rag^+/-^* and wild-type zebrafish (207). Sixteen proviral insertion sites in Moloney murine leukemia virus (PIM) kinases were recently found to have increased gene expression following SVCV infection in adult zebrafish kidneys at 24 hpi using RNA-Seq, and that three pan-PIM kinase inhibitors blocked viral entry (231). As several zebrafish fluorescent reporter strains have been used for FACS to isolate macrophages or neutrophils for cell-specific functional analysis (38, 48), RNA-Seq could be applied to characterize these FACS sorted cell populations following virus infection. Single cell RNA-Seq (scRNA-Seq) has been applied to study embryonic development (232) and tissue regeneration (233) in the zebrafish. This technology should prove valuable in characterizing the inflammatory response to viral infection and potentially identify genes that differentiate phagocytes between various states of activation.

## Roles of Non-Coding RNA

Genes function together in complex networks with multiple layers of genetic regulation that include both protein coding and non-protein coding genes. In the Ensembl annotation of the zebrafish genome [Ensembl version 103 annotation of GRCz11 (234)], there are 25,592 protein-coding genes, 3,227 small non-coding, and 3,278 long non-coding genes. These non-coding genes lack long open reading frames, and map to intergenic regions, introns, or antisense to protein-coding genes. Studies of non-coding genes in human and mouse have demonstrated important *cis*- and/or *trans*-regulatory roles in immune function as summarized below.

Long non-coding RNAs (lncRNAs) have transcripts that exceed 200 bp, and are classified based on their genomic location and orientation. Classes of lncRNAs include long intergenic RNA (lincRNA), antisense, bidirectional, intronic, and enhancer-associated RNAs. Diverse functions of lncRNAs have been described. They can function as both positive and negative regulators at the DNA, RNA or protein level in *cis* and *trans*. Some lncRNAs function in the nucleus to interact with chromatin, while others interact with RNAs or proteins in the cytoplasm. An example of a *cis*-regulatory lncRNA is the mouse antisense lncRNA, *Gm14023* (235). *Gm14023*, is antisense to *Il1a* and functions to regulate the recruitment of RNA polymerase II to the *Il1a* promoter following TLR ligand stimulation (235). Examples of *trans*-regulatory lncRNAs include the mouse antisense lncRNA, *Ttc39aos1*, that was originally named, *lncRNA-EPS* (236). A mouse knockout of *Ttc39aos1* and gain-of-function experiments showed that it was required to control the expression of immune response genes in macrophages (236). An example of a lncRNA that has been shown to function in both *cis* and *trans* is the mouse long intergenic RNA, *Ptgs2os2*, that was originally named, *lncRNA-Cox2* (237). Knockdown of *Ptgs2os2* by shRNA showed that the expression of proinflammatory genes (including *Tlr1*, *Il6*, and *Il23a*) was decreased, and chemokines (*Ccl5* and *Cx3cl1*), chemokine receptors (including *Ccr1*), and interferon-stimulated genes (including *Irf7*, *Oas1a*, *Oas1l*, *Oas2*, *Ifi204* and *Isg15*) were upregulated (237).

A study of the role of the adaptive immune system in response to SVCV in zebrafish kidneys found that 12,165 putative lncRNAs were expressed (207). The study examined lncRNA candidates by looking for differentially expressed protein coding genes that mapped to within 10 kbp of the lncRNA and testing for enriched Gene Ontology terms. Among putative lncRNAs investigated were two lncRNAs that map adjacent to *rag1* and *rag2* in the zebrafish genome, suggesting a regulatory role.

MicroRNAs are negative regulators of gene expression that have been shown to be required for zebrafish immune function (49, 148, 149, 238) in addition to embryonic development (239), and tissue regeneration (240, 241). Downregulation of both miR-722 (148, 149) and miR-199 (150) have been shown to be required for neutrophil migration in zebrafish. Studies of zebrafish with systemic *Pseudomonas aeruginosa* PAK strain infection showed that neutrophil expression of miR-722 was required for regulating the inflammatory response through Rac2 (149). Overexpression of miR-722 in the *Tg(lyz:mir722-Dendra2)^pu6^* line had increased survival to lethal inflammation caused by acute *Pseudomonas* infection. A screen of several microRNAs showed that miR-199-3a was required for neutrophil migration (150). Using the neutrophil-specific overexpression line, *Tg(lyz:mir722-Dendra2)^pu19^*, it was shown that miR-199 regulates cyclin-dependent kinase 2 (*cdk2*). Hypermaturation of neutrophils and defective interferon signaling was observed in miR-142a and miR-142b double-knockout zebrafish (49). Genes differentially expressed in miR-142 double-knockout included *stat1a* and *irf1b*. The neutrophil inflammatory response to tailfin injury was shown to be regulated by miR-223 by regulating nuclear factor (NFκB) signaling (238). Using miR-223 knockout and multiple miR-223 transgenic lines, it was shown the expression from both neutrophils and the basal and apical epithelium functioned to negatively regulate neutrophil recruitment. NFκB activity, visualized using the *Tg(6xHsa.NFκB : EGFP)^nc1^* line, was upregulated following tailfin injury in miR-223 mutants. The contribution of miR-233 expression in neutrophils was studied using the *Tg(lyz:RFP-mir223)^pu9^* along with a transgenic line that expressed a miR-223 sponge in apical epithelial cells, *Tg(krt4:RFP-bsmir223)^pu12^*. Specific miR-223 targets identified included *cul1a, cul1b, traf6*, and *tab1*.

**Figure 3:**
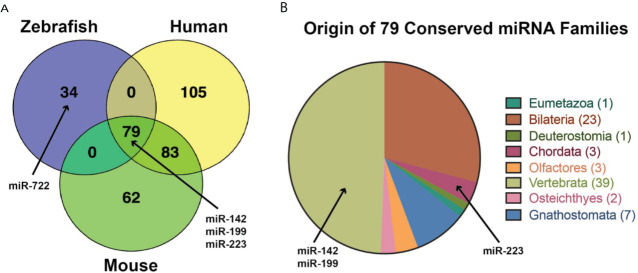
Overlap among miRNA families in zebrafish, mouse, and human genomes. **(A)** 79 miRNA families are conserved among zebrafish, mouse and human genomes, including miR-142, miR-199 and miR-223. 34 miRNA families are found in the zebrafish, but not in the mouse or human genome. One of the 34 miRNA families is miR-722 which was shown to regulate zebrafish neutrophil migration. 62 miRNA families are found in the mouse, but not in the zebrafish or human genome. 105 miRNA families are found in the human genome, but not in the zebrafish or mouse genome. 83 miRNA families are conserved between the mouse and human genomes that are not found in the zebrafish genome. **(B)** The origin of the 79 conserved miRNA families are labeled by the last common ancestor for Eumetazoa, Bilateria, Deuterostomia, Chordata, Olfactores, Vertebrata, Osteichthyes, and Gnathostomata with the number of families shown in parentheses. Two of the 79 miRNAs are miR-199 and miR-223 that have roles in neutrophil function. The node of origin for miR-142 and miR-199 is Vertebrata, and Gnathostomata for miR-223.

MiRNAs are important candidate genes to study in the inflammatory response to virus infection, but miRNAs conserved with humans should be prioritized. MiRNAs are highly conserved across animal taxa in an evolutionary context (242). One of the first miRNAs discovered, let-7, is conserved across metazoa, but other miRNAs, such as miR-722, are only found in teleost fish. MiRNAs are organized into families based on their seed sequence that is used to determine targets. Once a miRNA family evolves, it is rarely lost during evolution. As described in MiRGeneDB (243), the roundworm (*C. elegans*) has 145 miRNAs in 90 families, the zebrafish has 390 miRNAs in 113 families, the mouse has 447 miRNAs in 224, and humans have 556 miRNAs in 267 families. The number of miRNA families correlate with complexity that is estimated by the number of distinct cell types (242). In addition, the complexity of immune systems across metazoa correlates with the number of miRNA families. Studies of miRNAs in the response to viral infection in the zebrafish are promising as a total of 79 families, including miR-199, are conserved between zebrafish and humans (**Figure 3**).

## Discussion

Modeling viral infection in the zebrafish and other fishes have provided valuable information about the inflammatory response and other host-virus interactions that are complementary to other model systems. Zebrafish models of viral infection take advantage of the strengths of the model that include genetic tools and reporter lines that allow for *in vivo* imaging. One aspect of the inflammatory response to viral infection that needs additional study is the contribution of neutrophils. As summarized in this review, several existing zebrafish models have been designed to study neutrophil function. Some of these tools have begun to be the applied to study viral infection as the role of the inflammatory response of neutrophils during viral infection is largely unknown.

**Figure 4:**
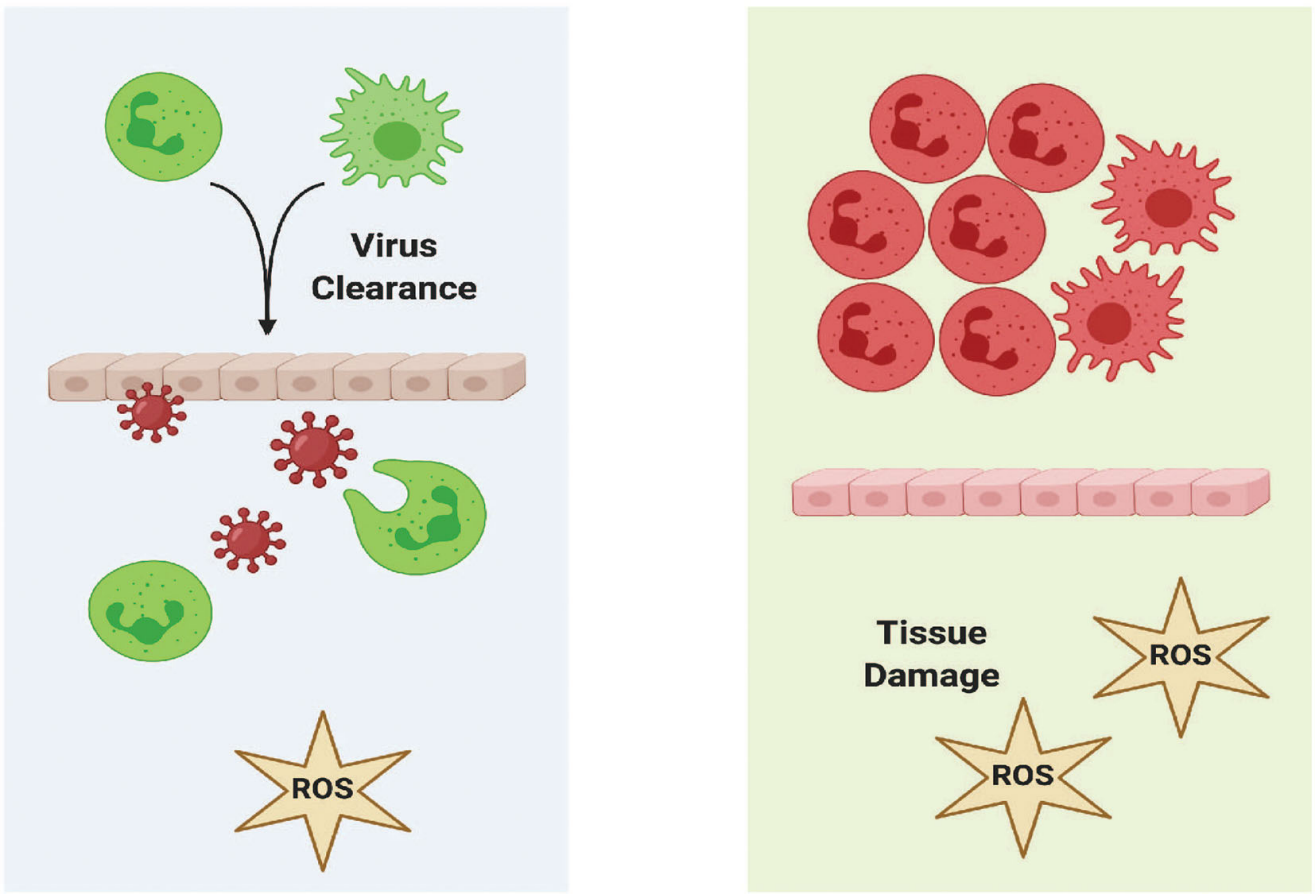
Immunological Tipping Point in The Inflammatory Response to Virus Infection. A balance between the role of antiviral and hyperinflammatory responses by neutrophils must be maintained to avoid tissue damage during infection by IAV or other viruses. We hypothesize that the modulation of ROS is a central factor in regulating the response to virus infection.

We hypothesize that there is an immunologic tipping point during viral infection between the beneficial antiviral activity and tissue damaging hyperinflammatory response of neutrophils (**Figure 4**). ROS generated by virus-infected cells may initiate neutrophil chemotaxis during an IAV infection. By recruiting neutrophils to areas of virus-induced tissue damage through the formation of H_2_O_2_ gradients, these neutrophils may then be retained at the site because the high ROS levels suppress cell motility. ROS play critical roles in the immune response, serving both as indicators of immune dysregulation and as mediators of various immune processes, including neutrophil migration. The roles of ROS in viral infection have not been definitively identified. In addition, type I and type II IFN together reduce neutrophil migration and limit hyperinflammation during IAV infection. The connections linking the effectors of ROS production, however, like the NADPH oxidase and myeloperoxidase, as well as the mechanisms driving the suppression of neutrophil migration by interferon signaling, are unknown. Our hypothesis is that neutrophils, while controlling an IAV infection, trigger excessive inflammation through mechanisms involving ROS production and type I IFN signaling.

The importance of neutrophils in the innate response to viral infection is an ongoing subject of controversy. Zebrafish models of virus infection are uniquely poised to enable characterization of the molecular signals that stimulate neutrophils to migrate *in vivo* and elucidate pathways that lead to generation of ROS and other mediators of inflammation in the antiviral response. Furthermore, studies that model human viruses in zebrafish, such as IAV, have the potential to provide unique insight regulating neutrophil function during the inflammatory and antiviral responses. One advantage of the zebrafish model is the potential to screen small molecules to identify potential candidate therapeutics at relatively low cost. One example was demonstrating that the neuraminidase inhibitor, Zanamivir, extended survival in our zebrafish model of IAV infection (8). These advances may inform the development of new treatments that modulate the inflammatory response to viruses like IAV.

## Author Contributions

CS and BK developed the overall outline of the manuscript. B-LS created all tables and **Figures 1**, **2** and **4**. BK created **Figure 3**. CS, B-LS, CK, PM and BK wrote the manuscript. All authors contributed to the article and approved the submitted version.

## Funding

The authors were supported by the National Institute of Allergy and Infectious Diseases of the National Institutes of Health under grant number R15 AI131202. BK was also supported by an Institutional Development Award (IDeA) from the National Institute of General Medical Sciences of the National Institutes of Health under grant number P20 GM103423. The funding sources played no role in the design of the study, in collection, analysis, and interpretation of data, or in writing the manuscript.

## Conflict of Interest

The authors declare that the research was conducted in the absence of any commercial or financial relationships that could be construed as a potential conflict of interest.
